# Geographical and temporal variations of serogroups and clonal types of Neisseria meningitidis involved in culture-confirmed invasive meningococcal disease in Canada, 2015–2023

**DOI:** 10.1099/jmm.0.001979

**Published:** 2025-03-12

**Authors:** Courtney Meilleur, Jianwei Zhou, Linda Hoang, Gregory Tyrrell, Jessica Minion, Paul Van Caeseele, Julianne Kus, Brigitte Lefebvre, David Haldane, Richard Garceau, George Zahariadis, Xiaofeng Ding, Kami Kandola, Sudit Ranade, Raymond S. W. Tsang

**Affiliations:** 1 Vaccine Preventable Bacterial Diseases, Science, Reference and Surveillance Directorate, National Microbiology Laboratory Branch, Pubic Health Agency of Canada, Winnipeg, Manitoba, Canada; 2 British Columbia Centre for Disease Control, Vancouver, British Columbia, Canada; 3 Provincial Laboratory for Public Health, Edmonton, Alberta, Canada; 4 Roy Romanow Provincial Laboratory, Regina, Saskatchewan, Canada; 5 Cadham Provincial Laboratory, Winnipeg, Manitoba, Canada; 6 Public Health Ontario Laboratory, Toronto, Ontario, Canada; 7 Laboratoire de santé publique du Québec, Institut nationale de sante publique du Québec, Sainte-Anne-de-Bellevue, Québec, Canada; 8 Pathology and Laboratory Medicine Program, Nova Scotia Health Authority, Halifax, Nova Scotia, Canada; 9 Provincial Health Laboratory, Moncton, New Brunswick, Canada; 10 Newfoundland and Labrador Public Health Laboratory, St. John's, Newfoundland and Labrador, Canada; 11 Provincial Laboratory Services, Government of Prince Edward Island, Charlottetown, Prince Edward Island, Canada; 12 Northwest Territories Health and Social Services Authority, Government of Northwest Territories,, Yellowknife, Northwest Territories, Canada; 13 Health and Social Services, Government of Yukon, Whitehorse, Yukon, Canada

**Keywords:** clonal analysis, invasive meningococcal disease, serogroup, temporal and geographical variations

## Abstract

**Introduction.** Invasive meningococcal disease (IMD) is a nationally notifiable illness in Canada due to its potential severity and transmissibility. Vaccination strategies differ by province/territory and are informed by changes in the antigenic characteristics of circulating strains.

**Gap Statement.** Though IMD statistics are tracked at a provincial/territorial level, there is a lack of published data characterizing trends in the epidemiology of this disease at a national level.

**Aim.** To examine the epidemiology of culture-confirmed IMD in Canada during the period of 2015–2023.

**Methodology.** Meningococcal isolates sent to the National Microbiology Laboratory Branch between 2015 and 2023 as part of routine national surveillance were characterized for serogroup by bacterial agglutination and genetic methods. Clonal analysis was done by MLST. Demographic information was derived from requisition forms accompanying the samples.

**Results.** The proportion of IMD caused by serogroup W meningococci (MenW) in 2015–2023 was 30.0% with more (62.9%) MenW cases detected in western provinces. Serogroup B meningococci (MenB) IMD was more common (53.4%) in Quebec and Atlantic Canada. Clonal analysis reveals 168 distinct sequence types between 2015 and 2023, with 103 belonging to MenB. The average age of MenB cases during this time was 29.1 years, significantly younger than serogroup C (MenC) (45.3 years), serogroup Y (MenY) (48.3 years) and MenW (43.0 years) patients. Additionally, 31.5% of MenB and 21.7% of MenC IMD isolates were collected from cerebrospinal fluid (CSF) or brain samples, which were significantly higher than that for MenY (12.2%) and MenW (7.3%) isolates.

**Conclusions.** Results from this and previous studies showed temporal and geographical variations in the serogroups causing IMD in Canada. MenB also showed the most genetic diversity, caused IMD in a significantly younger population and was more often isolated from CSF and brain samples than other serogroups.

## Introduction

Invasive meningococcal disease (IMD) is a rare but serious illness caused by the bacterium *Neisseria meningitidis*. In Canada, it is a significant nationally notifiable infectious disease despite its low incidence [1]; during the period of 2012–2019, there was a yearly average of only 120 cases [2]. However, the relatively high case fatality rate (e.g. about 13.8% during the period of 2012–2019), serious sequelae [3] coupled with the potential of causing localized outbreaks or epidemic spread make this disease a high priority for public health. *N. meningitidis* can be classified as belonging to 1 of 12 serogroups (A, B, C, E, H, I, K, L, W, X, Y and Z), but most disease-causing strains belong to one of six serogroups: A, B, C, W, X and Y (MenA, MenB, MenC, MenW, MenX and MenY) [4].

In Canada, surveillance of IMD was established in 1924 making IMD a nationally notifiable disease, and data has been captured in the Canadian Notifiable Diseases Surveillance System which can be viewed online (https://diseases.canada.ca/notifiable/charts?c=pl). However, only limited epidemiological information is captured. In 1992, an Enhanced Invasive Meningococcal Disease Surveillance System was established that linked provincial/territorial epidemiological information and national laboratory data, and summaries are periodically published (5; Saboui *et al*. 2022), but the focus is not on the bacterial characterization. The National Microbiology Laboratory (NML) of the Public Health Agency of Canada works closely and collaboratively with provincial and territorial public health laboratories to provide a national laboratory surveillance programme on IMD. In this programme, invasive isolates from all culture-confirmed IMD patients are routinely submitted by the provincial and territorial public health laboratories to the NML. Approximately 79% of IMD cases reported by provinces and territories have isolates submitted to the NML (Saboui *et al*. 2022). The remaining cases were either diagnosed by PCR methods without a positive bacterial culture, or the isolates had been lost such as not preserved for passing on to either provincial public health laboratories or the NML. Over the years, this programme has published a number of observations on *N. meningitidis* strain characteristics [6,11].

Historical data suggests the prevalence of disease, and serogroup distribution may vary temporally and geographically, with some serogroups being more prevalent in certain regions than others. For example, we have described that higher percentages of IMD were due to MenB in the province of Quebec as well as in the Atlantic provinces when compared to Western Canada [12, 10]. This variation is further affected by the introduction of meningococcal vaccine programmes [13], which may also differ from region to region. Furthermore, new strains may emerge with greater potential to cause outbreaks [14]. In Canada, the emergence of a virulent clone of MenC electrophoretic type 15 [or sequence type (ST) 11 clonal complex (CC)] caused localized and province-wide outbreaks involving multiple jurisdictions [15]. Subsequently, in 2003, a new virulent MenB strain characterized by the antigenic formula of B:17:P1.19 and ST-269 CC emerged in the province of Quebec and caused a prolonged outbreak in the province [16, 12]. Consequently, standardized methods of surveillance including characterizations of strains have been established in order to track these changes nationally, globally and over time[17]. *N. meningitidis* serogroups can be classified phenotypically by detection of surface carbohydrate capsule antigens using antisera and/or genetically by identifying their serogroup-specific capsule synthesis genes. Though the carbohydrate serogrouping antigen is one of the most important virulence factors (and also a protective antigen), certain *N. meningitidis* isolates may also belong to particular virulent strains called hypervirulent clones or STs [18, 19] Clonal analysis of *N. meningitidis* is done by nucleotide sequencing of seven housekeeping enzyme genes (multi-locus sequence typing or [MLST]) [20] and isolates are characterized by their combination of housekeeping gene alleles. Different allelic profiles give rise to different STs and related STs are grouped together as CC.

Control of MenA, MenC, MenW, and MenY IMD can be afforded by the polysaccharide-based conjugated vaccines such as the monovalent MenC- or the quadrivalent MenACWY-conjugated vaccines which induce bactericidal antibodies directed against the surface capsular antigens of these four serogroups of meningococci [21]. However, the capsule of MenB is poorly immunogenic due to cross-reactivity with the human neural cell adhesion molecule [22]. Therefore, protein-based vaccines such as the 4CMenB (Bexsero) [23] or the bivalent factor H-binding protein (Trumenba^®^) vaccines [24] have been developed for the protection of MenB IMD.

In Canada, MenC conjugate vaccine was first used in 2001 to control a MenC outbreak in the province of Quebec [25]. Soon after, the Canadian National Advisory Committee on Immunization released a first statement regarding the use of the recently licensed MenC conjugate vaccine [26]. All provinces and territories now have routine immunization programmes for infants against MenC IMD. An adolescent booster dose with either monovalent MenC-conjugated vaccine or quadrivalent MenACWY-conjugate vaccine is usually provided in programmes delivered in high schools. However, the dosage and schedule used at the provincial and territorial levels vary across the country [27]. In addition, the timing of adoption of these vaccination programmes and the choice of vaccine (monovalent C- or quadrivalent MenACWY-conjugated vaccines) also vary across the country depending on the local epidemiology (reviewed in [25]). A table showing the current provincial and territorial routine and catch-up meningococcal vaccination schedule for infants and children in Canada is available online from the Public Health Agency of Canada website [28].

A previous study based on culture-confirmed IMD case isolates received between 2010 and 2014 showed that 63.5% of the IMD cases in Canada were due to serogroup B (MenB), 22.7% were due to MenY and other serogroups like MenC and MenW were not common (responsible for 7.5 and 1.5% of IMD, respectively) [29]. In this study, we report on the serogroup distribution of culture-confirmed IMD cases by province over the period of 2015–2023. We also analyse the epidemiology of IMD cases based on age and gender information as well as by isolation sites that yielded positive culture. Finally, we examine the clonal types of *N. meningitidis* responsible for causing IMD in Canada during this period.

## Methods

### Bacterial isolates

Invasive *N. meningitidis* isolates are routinely sent to the National Microbiology Laboratory Branch (NMLB) by provincial and territorial public health laboratories for serogrouping or confirmation and additional typing as part of a national IMD surveillance programme. Between 1 January 2015 and 31 December 2023, a total of 774 culture-confirmed invasive *N. meningitidis* isolates were sent to the NML for characterization, with each specimen representing a different infection. Isolates sent from the same patient within 1 month were considered to be likely the same infection and as such excluded from analysis. Pure culture prepared from a sub-culture of the primary specimen was preserved in brain heart infusion broth with 20% glycerol for long-term storage at −80 °C in cryovials. Patient demographics attendant to each case were taken from the requisition form that accompanied each isolate sent to the NMLB.

### Canadian provinces, territories and population

Population estimates for Canada (current population 41.3 million as of 1 July 2024) as well as each province and territory were retrieved from Statistics Canada for 2015–2023 [30]. A map of Canada has been included to communicate the geographical relationships of the various provinces and territories (Fig. S1, available in the online Supplementary Material).

### Serogrouping

Serogrouping of isolates by bacterial slide agglutination was done using group-specific rabbit antiserum, produced in-house. To produce the antisera, rabbits were inoculated intravenously with live *N. meningitidis* cells of differing serogroups and allowed to develop a specific antibody response over 5 weeks, at which point the animals were sacrificed and the antiserum was collected. Autoagglutinable and otherwise non-groupable strains were characterized using PCR, which was also used to confirm grouping assigned by agglutination results [16]. To identify the most common serogroups of isolates using PCR, we used the following primers: serogroup B (forward 5′-CTC TCA CCC TCA ACC CAA TGT C-3′, reverse 5′-TGT CGG CGG AAT AGT AAT AAT GTT-3′), serogroup C (forward 5′-GCA CAT TCA GGC GGG ATT AG-3′, reverse 5′-TCT CTT GTT GGG CTG TAT GGT GTA-3′), serogroup Y (forward 5′-CTA ATC ATG ACA TCT CAA AGC GAA GGC-3′, reverse 5′-TTA AAG CTG CGC GGA AGA ATA GTG AAA T-3′) and serogroup W (forward 5′-TGA TCA TGA CAT CAG AAA GTG AGG GAT T-3′), which shares the same reverse primer as serogroup Y.

Positive and negative controls for both the agglutination test and PCR included either pure cultures or crude heat lysates of known serogroups as positive controls, whilst sterile PBS was used as negative controls for both methods.

### MLST and whole-genome sequencing (WGS)

Isolates were further characterized based on gene sequences of their seven different housekeeping genes and assigned an ST according to their resulting MLST profile [20]. DNA for MLST analysis was obtained from pure cultures grown overnight at 37 °C on 5% sheep blood agar, and a simple preparation of boiled lysate was sufficient to liberate enough template for PCR amplification as previously described [16].

MLST profiles for isolates were either obtained by PCR sequencing of seven housekeeping genes (https://pubmlst.org/organisms/neisseria-spp) or derived from WGS data. For WGS, genomic DNA was extracted using an Epicentre MasterPure Complete DNA and RNA extraction kit (Mandel Scientific, Guelph, ON, Canada) prior to library preparation and sequencing. The Illumina MiSeq platform (Illumina, San Diego, CA, USA) was used to generate 2×300 bp sequences with MiSeq reagent kit v3 and TruSeq library preparation; genomes were then assembled using the Integrated Rapid Infectious Disease Analysis tool [31]. MLST alleles and the STs were identified from the WGS data using the Genome Comparator plugin on the Bacterial Isolate Genome Sequence Database (BIGSdb) [32]. Assignments of STs and CCs were according to the Neisseria PubMLST website (https://pubmlst.org/organisms/neisseria-spp).

The genetic diversity of serogroups was compared by dividing the number of isolates per serogroup by the number of STs identified in that serogroup. The smaller the number, the more number of STs found in the serogroup, reflecting the degree of diversity.

### Statistical analysis

Odds ratio [33] was used to study the following association of serogroups over time, or by province, or association with CSF/brain or joint isolation. Statistical differences between groups were evaluated using GraphPad Prism 5 for Windows, version 5.03 [34]. To compare isolate counts per serogroup between provinces and between different years, the Kruskal–Wallis analysis of variance test (*α*=0.05) was used to evaluate the difference between mean values. Post-hoc Dunn’s test was used to evaluate specific differences between statistical groups, with Sidak’s correction applied to account for multiple comparisons between groups. For comparisons in which a specific *P*-value was reported, an online calculator based on the R statistics package [35] was used to validate calculations performed in Microsoft Excel [36].

## Results

### Temporal and geographical variations in culture-confirmed invasive meningococcal disease cases by serogroups

**Fig. 1.**
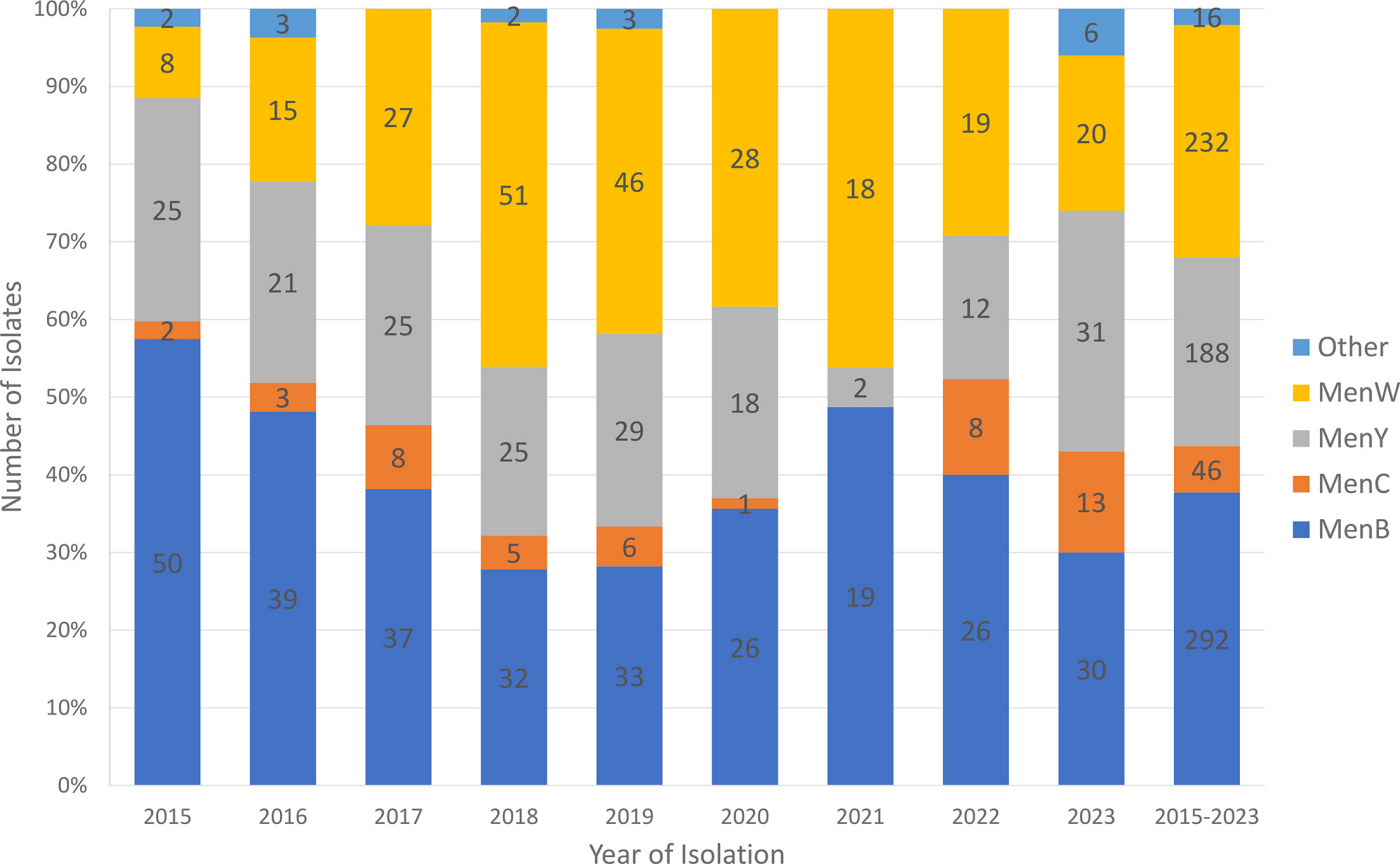
Temporal distribution of serogroups of *N. meningitidis* from culture-confirmed invasive meningococcal disease cases in Canada, 2015–2023.

A total of 774 individual cases occurred in the 9-year period with an average of 86 cases per year. Of the 774 invasive *N. meningitidis* received for typing, 762 have a serogroup assigned by bacterial agglutination test with serogroup antisera, and 12 were non-groupable by this method. Of these 12 non-groupable isolates, one was identified as MenY by PCR, whilst the remaining 11 isolates were found to be non-encapsulated strains based on the presence of the *crgA* gene but the absence of the *ctrA* gene. The number of cases per year ranged from a low of 39 cases in 2021 (pandemic year) to a high of 117 cases in 2019 (pre-pandemic) (Fig. 1). IMD case numbers in 2020 (*n*=73 of which 26 were MenB) and 2021 (*n*=39 of which 19 were MenB) (SARS-CoV-2 pandemic years) were noticeably lower compared with other years, increasing in 2022 (*n*=65 of which 26 were MenB); until by 2023 (*n*=100 of which 30 were MenB), the number of IMD cases reached almost the pre-pandemic levels. Of the 774 IMD cases, MenB was responsible for 292 cases (37.7%), followed by MenW (232 cases or 30.0%), MenY (188 cases or 24.3%) and MenC (46 cases or 5.9%). There were also 3 MenE cases, 2 MenZ cases and 11 cases due to non-encapsulated *N. meningitidis* strains.

At the beginning of the study period in 2015, MenB accounted for more than half (57.5%) of all IMD cases, followed by MenY (28.7%); both MenW and MenC were not common, each accounting for less than 10.0% of IMD cases. However, by 2023, MenY was responsible for 31.0% of all IMD cases, followed by MenB (30.0%), MenW (20.0%) and MenC (13.0%), whilst another 6% were due to either serogroup Z (one case) or non-encapsulated strains (five cases) (Table S1). When compared to the numbers in 2015, the number of MenW IMD was significantly higher in 2017–2023 (odds ratio-derived *P*-values ranged from 0.0434 in 2023 to <0.0001 in 2018–2021; *P*-values of 0.0021 and 0.0023 for 2017 and 2022, respectively).

The increase in MenW IMD began in 2016 (18.5% of all IMD cases), increasing gradually year after year until 2021 when nearly half (46.2%) of all IMD cases were due to MenW (Fig. 1). This increase in MenW disease did not affect all provinces equally, becoming less pronounced in the eastern part of the country (Table S2). The percentages of IMD cases due to MenW in British Columbia, Alberta or Manitoba were all significantly higher than that in Ontario, Quebec or Atlantic provinces (*P*-values <0.0001 as assessed by the Kruskal–Wallis test with post-hoc Dunn’s comparison). The percentage of IMD cases due to MenW in Saskatchewan was higher when compared to that in Ontario, but it did not reach statistical significance (*P*-value=0.0584), whilst it was statistically higher when compared to that in Quebec or Atlantic Canada (*P*-values for each comparison were both 0.0002). In three Western provinces (British Columbia, Alberta and Manitoba), MenW was responsible for >50% of all IMD cases for the overall period 2015–2023 (Table S2). During this same period, MenW was responsible for 47.1, 25.2 and 10.7% of all culture-confirmed IMD cases in Saskatchewan, Ontario and Quebec, respectively, whilst in Atlantic Canada, only four cases (5.9%) of MenW IMD were noted.

Temporally, in British Columbia, MenW was responsible for 60.9, 69.6, 77.8 and 63.6% of IMD in 2017, 2018, 2019 and 2020, respectively (Table S3). In Alberta, 50.0, 57.9, 66.7, 90.0, 100 and 80.0% of IMD were also due to MenW from 2017 to 2022. For Saskatchewan and Manitoba, there were only one to three MenW cases per year in each province recorded during the period from 2015 to 2022, but in 2020 for Saskatchewan, MenW was responsible for 75.0% (three cases) of IMD, whilst in Manitoba for 2023, ten MenW cases occurred accounting for 76.9% of IMD in that year. In contrast, the increase in invasive MenW disease in Central and Eastern Canada was much smaller in scale and occurred in 2018 and 2019. For example, in Ontario, 35.5% (11 cases) and 34.4% (11 cases) of IMD in 2018 and 2019, respectively, were due to MenW; in Quebec, 28.0% (seven cases) of IMD were due to MenW in 2018; whilst in Atlantic Canada, only three cases of MenW were recorded in 2019 accounting for 27.3% of IMD in that region.

**Fig. 2.**
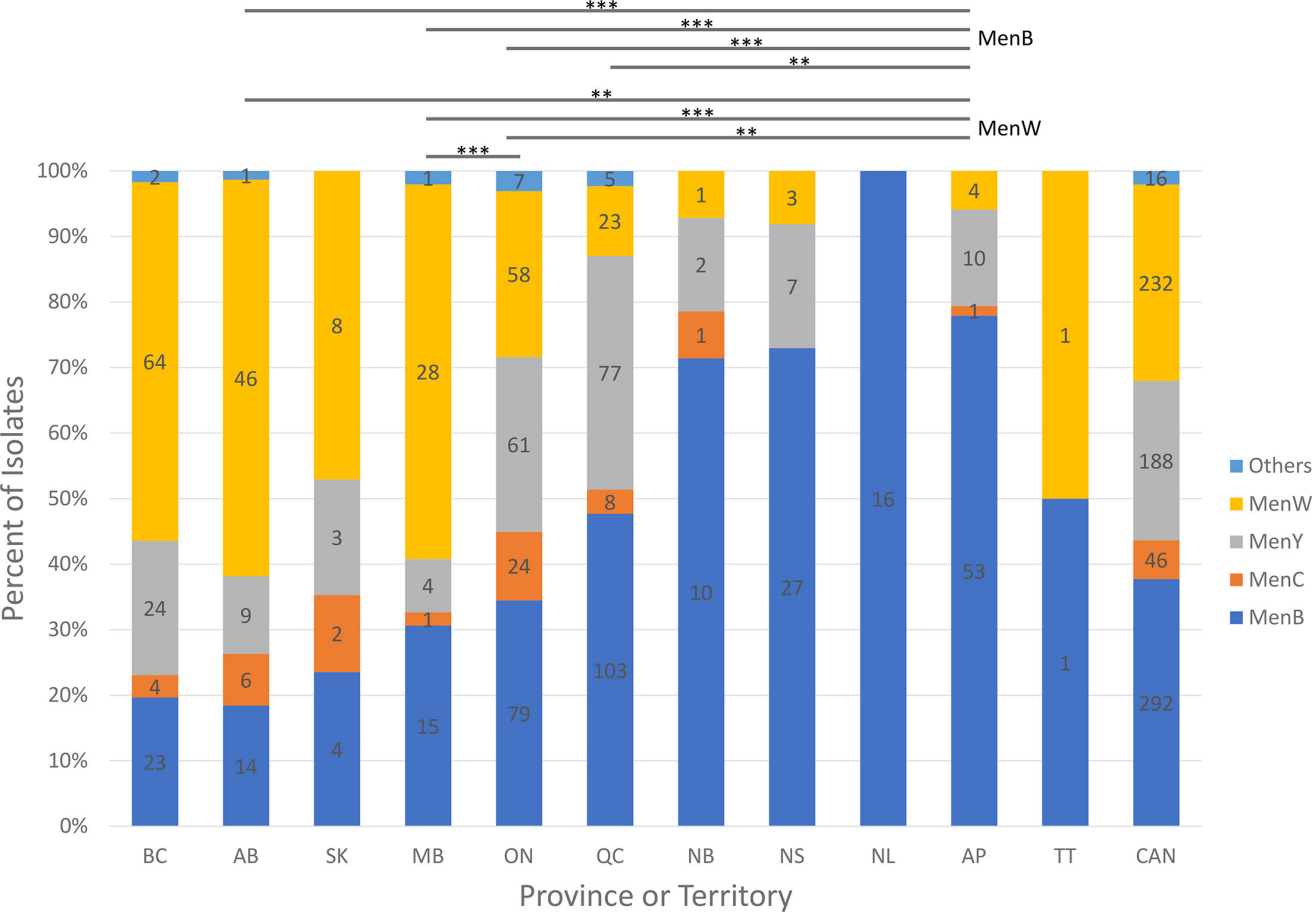
Geographical variations of culture-confirmed invasive meningococcal serogroups^^^ in Canada^+^, 2015–2023. ^^^Serogroup B (MenB), serogroup C (MenC), serogroup W (MenW) and serogroup Y (MenY). ^+^Canadian provinces and territories: British Columbia (BC), Alberta (AB), Saskatchewan (SK), Manitoba (MB), Ontario (ON), Quebec (QC), New Brunswick (NB), Nova Scotia (NS), Newfoundland and Labrador (NL), Atlantic provinces (AP) (comprise of New Brunswick, Nova Scotia, Newfoundland and Labrador and Prince Edward Island), Northern Territories (TT) (consists of one MenW from Yukon and one MenB from Northwest Territories) and Canada (CAN). Asterisks denote significance values as assessed using a Kruskal–Wallis test with post-hoc Dunn’s test and Sidak’s correction, where *=*P*≤0.05, **=*P*≤0.01 and ***=*P*≤0.001.

In contrast to the concentration of MenW IMD in Western Canada in the period of 2015–2023, in both Quebec and Atlantic Canada, there was a statically higher percentage of IMD identified as MenB (47.7% in Quebec and 77.9% in Atlantic Canada) than in the rest of Canada (34.5% in Ontario, 30.6% in Manitoba, 18.4% in Alberta and 19.7% in British Columbia) except Saskatchewan (23.5%) when compared with Quebec (by the Kruskal–Wallis test, *P*-value=0.0647 whilst *P*<0.0001 for comparison with British Columbia or Alberta, and *P*-values for comparison with Manitoba or Ontario were 0.00321 and 0.0048, respectively). In Atlantic Canada, the percentage of IMD identified as MenB was significantly higher than in every other province including Quebec (*P*-values were all <0.0001) (Fig. 2, Table S2). In the province of Quebec, although there was an overall more MenB than any other serogroups as a cause of IMD, temporally, the percentage of MenB as a cause of IMD decreased over time from 84.0% in 2015 to 25.0% in 2023, whilst the percentage of MenY as a cause of IMD increased from 8.0% in 2015 and gradually to 47.4% in 2022 and 55.0% in 2023 (Table S3).

During the period 2015–2023, of all the Canadian MenY IMD cases, 41.0, 32.5 and 12.8% were found in the provinces of Quebec, Ontario and British Columbia, respectively, cumulating to a total of 86.2% of all MenY cases (Table S2). In the provinces of Quebec, Ontario and British Columbia, MenY were responsible for 35.6, 26.5, and 20.5% of IMD there, respectively. Only 16 (or 8.5%) of the Canadian total MenY IMD cases were detected in the western and prairie provinces of Alberta, Saskatchewan and Manitoba. Ten (5.3%) of the Canadian MenY IMD were found in the Atlantic provinces of New Brunswick, Nova Scotia, Newfoundland and Labrador and Prince Edward Island.

Over half (24 cases or 52.2%) of the MenC cases were in Ontario. Ten (21.7%) of MenC cases were in the western provinces of British Columbia and Alberta, eight cases (17.4%) were in Quebec, three cases (6.5%) were in the prairie provinces of Saskatchewan and Manitoba and only one case (2.2%) occurred in the Atlantic provinces of New Brunswick.

### Clonal analysis of invasive *N. meningitidis*

The Canadian invasive MenW case isolates (*n*=232) were highly clonal with 94.0% (*n*=218) belonging to the ST-11 CC and 208 (95.4%) typed as a single ST, ST-11. The remaining ten isolates were divided into nine different but related STs. In contrast, the 11 ST-22 CC isolates belonged to seven different but related STs (Table S4D). The remaining three MenW isolates belonged to three different STs: ST-11739 (ST-60 CC), ST-15377 (ST-9316 CC) and ST-1308 (unassigned to any known CC). The 20 STs identified in the 232 MenW isolates can be found listed in Table S4D.

Nearly three quarters (73.9% or 139 isolates) of the 188 invasive MenY were typed as ST-23 CC with 83 isolates (59.7%) belonging to ST-23; 28 isolates (20.1%) belonged to ST-1655, 8 isolates belonged to ST-3582, 5 isolates belonged to ST-10466, 5 isolates belonged to ST-10732, 2 isolates belonged to ST-3587 and the rest (eight isolates) belonged to eight different but related STs. Other important CCs included 27 isolates of ST-167 CC and another 6 isolates typed as ST-174 CC. All six ST-174 CC isolates were typed as ST-1466; four were found in the central (Ontario) and two in eastern (Quebec) provinces, which were spread over the study period of 2015 (one isolate), 2017 (one isolate), 2018 (one isolate) and 2023 (three isolates). Five other STs were unassigned to any known CC. The 32 STs associated with MenY in Canada can be found in Table S4C. Invasive MenY strains were significantly more clonally diverse as compared to MenC (*P*=0.0019) and MenW (*P*=0.0039 as assessed using Dunn’s comparison after the Kruskal–Wallis test) (Table 1).

**Table 1.** Clonal analysis by MLST of invasive *N. meningitidis* serogroup B (MenB), serogroup C (MenC), serogroup Y (MenY) and serogroup W (MenW) in Canada, 2015–2023. Isolates were characterized by STs, and related STs were grouped together as CCs

Characteristics	MenB	MenC	MenY	MenW
No. of case isolates	292	46	188	232
No. of CCs	17	6	7	4
No. of ST not assigned to any known CC	15	3	5	1
Total no. of STs	103	13	32	20
Diversity index (no. of isolates per ST)	2.83	3.54	5.88	11.60

When compared to invasive MenW, the Canadian invasive MenC cases (like MenY) were relatively more diverse with 71.7% (33 isolates) belonging to the ST-11 CC, and 31 of them were typed as ST-11; the remaining two isolates were typed as either ST-5752 or ST-12819 (both are single-locus variants of ST-11). There were three isolates each (6.5%) belonging to ST-269 CC or ST-35 CC, whilst the remaining seven isolates were typed as seven different STs grouped into three different CCs or did not belong to any known CC (Table S4B).

The Canadian invasive MenB isolates (*n*=292) were significantly more genetically diverse than MenC, MenY and MenW (*P*=0.0003 for each comparison by the Kruskal–Wallis test), with 103 STs identified (Table 1). Eighty-eight STs (270 isolates) were grouped into 17 different CCs, and the remaining 15 STs (22 isolates) did not belong to any known CC. The five most commonly encountered invasive MenB CCs were ST-41/44 CC (120 isolates or 41.1%), ST-269 CC (71 isolates or 24.3%), ST-32 CC (21 isolates or 7.2%), ST-213 CC (19 isolates or 6.5%) and ST-1157 CC (nine isolates or 3.1%), and together, they accounted for the majority of the invasive MenB isolates (*n*=240 or 82.2%). The genetic diversity of the four major meningococcal serogroups is summarized in Table 1 and Table S4A.

Overall, the ST-41/44 CC appeared to have a high degree of genetic diversity with 36 different STs found in 120 isolates. Two common STs, ST-154 (51 isolates or 42.5%) and ST-6617 (12 isolates or 10.0%), showed geographical concentrations. Forty-five (88.2%) of the ST-154 were found in Atlantic Canada (23 isolates), Manitoba (13 isolates) and Ontario (9 isolates), whilst rarely found (with only six isolates) in other provinces and territories. Similarly, 10 of the 12 ST-6617 isolates were found in Ontario, and the remaining two isolates were from British Columbia.

Of the 71 MenB case isolates of ST-269 CC, two STs (ST-269, *n*=44; ST-1161, *n*=10) accounted for 76.1% of the MenB in this CC. These two STs were also found mostly in two different eastern provinces: 39 of 44 (88.6%) ST-269 isolates were recovered in the province of Quebec, whilst seven of ten (70.0%) ST-1161 were found in the province of Newfoundland and Labrador. These two STs contributed to the finding that the majority of MenB Canadian ST-269 CC cases (67 of 71 or 94.4%) were found in Central and Eastern Canada, with most cases in Quebec (48 cases or 67.6%) followed by Atlantic Canada (11 cases or 15.5%) and Ontario (8 cases or 11.3%). There were only four (5.6%) MenB cases of ST-269 CC in Western Canada. Additionally, of the eight MenB cases identified to be caused by ST-60 CC, seven were found in the province of Newfoundland and Labrador. The full list of MenB STs can be found in Table S4A.

### Characteristics of Canadian IMD cases from 2015 to 2023 according to serogroups

Most MenB appeared to affect three age groups (those less than 5 years old made up of 30.5% of MenB cases; those over the age of 40, 31.9%; and those between 15 and 24 years old, 20.2%) (Table S6). Most MenC (80.4%) affected those aged 25 and above, whilst MenY affected more in those aged 40 and above (59.6%) and those between the ages of 15 and 24 years (24.5%). MenW appeared to affect the same three age groups as MenB but with higher percentages of MenW cases in those over the age of 40 years (56.1%) and less in those less than 5 years old (15.7%) or those between the ages of 15–24 years (13.5).

**Fig. 3.**
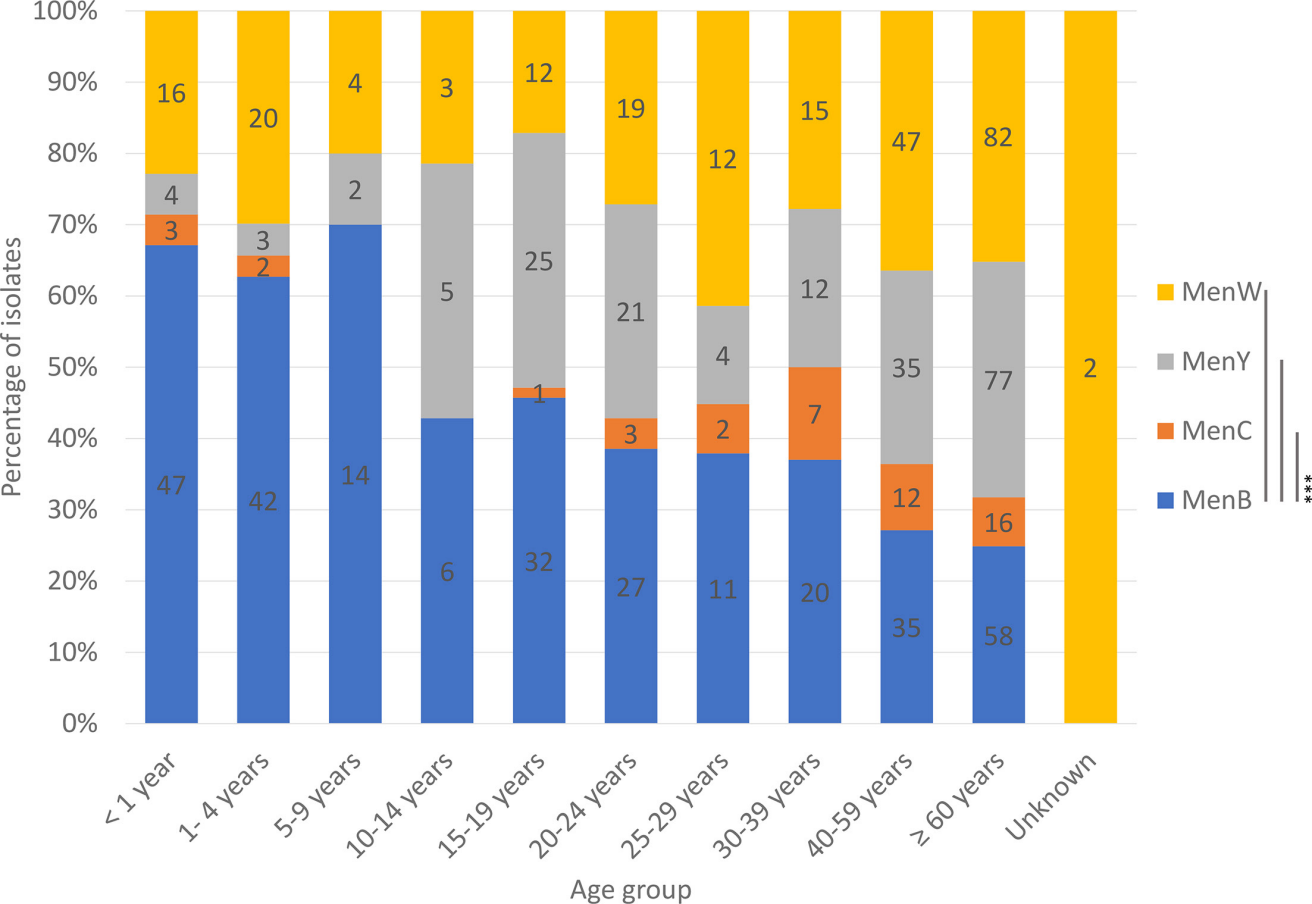
Age group distribution of culture-confirmed invasive meningococcal disease cases in Canada, 2015–2023 by serogroups^^^. ^^^Serogroup B (MenB), serogroup C (MenC), serogroup W (MenW) and serogroup Y (MenY). Asterisks denote significance values as assessed using a Kruskal–Wallis test with post-hoc Dunn’s test and Sidak’s correction, where *=*P*≤0.05, **=*P*≤0.01 and ***=*P*≤0.001.

The mean (29.1 years) and median (20.0 years) ages of MenB IMD were significantly (as determined using the Kruskal–Wallis test with Dunn post-hoc comparison) lower than that of MenC (45.3 years/48.0 years, *P*=0.00012), MenY (48.3 years/52.0 years, *P*<0.0001) and MenW (43.0 years/48.0 years, *P*<0.0001) IMD (Fig. 3). Also, 30.5% of MenB IMD occurred in those under the age of 5 years, whilst only 10.9, 3.7 and 15.7%, of MenC, MenY and MenW IMD, respectively, were in this age group. In contrast, the percentages of MenC, MenY and MenW IMD cases in those ≥60 years old were 34.8, 41.0 and 35.7%, respectively, whilst only 19.9% of MenB IMD were in this age group. Nearly a quarter (24.5%) of MenY and a fifth (20.2%) of MenB IMD were in those aged 15–24 years, whilst only 8.7% of MenC and 13.5% of MenW IMD were in this age group (Table S6).

Whilst nearly a third (30.5%) of MenB and slightly more than a fifth (21.7%) of MenC IMD cases were found to have *N. meningitidis* isolated from their CSF or brain [suggesting central nervous system (CNS) involvement], only 7.3 and 12.2% of MenW and MenY IMD cases showed CNS involvement (based on the source of isolation of submitted isolates). Between 2015 and 2023, odds ratio analysis shows that MenB was statistically more likely to be isolated from the CSF or brain than MenY (*P*=0.0065) or MenW (*P*<0.0001). Significantly higher percentages of MenC than MenW (*P*-value=0.0028) but not MenB nor MenY (*P*-values of 0.1832 or 0.1015, respectively) were recovered from CSF or brain. The percent of MenC isolated from joint or synovial fluid was significantly higher than that for MenB, MenY or MenW (*P*-values of 0.0013, 0.0120 and 0.0013, respectively) (Table S6).

## Discussion

This study highlighted the dynamic nature of IMD as evident from the temporal and geographical variations of serogroups and clonal types. During this study period, there was a shift in the serogroups responsible for IMD in Canada from mostly MenB (57.5% of all IMD cases) in 2015 to more MenY (31.0% MenY versus 30.0% MenB) in 2023. Also, an increase in the proportion of IMD due to MenW has increased from 9.2% in 2015 to 20% in 2023. The increase in MenW IMD occurred mostly and more pronouncedly in Western Canada: British Columbia (54.7% of IMD), Alberta (60.5%), Saskatchewan (47.1%) and Manitoba (57.1%). Furthermore, the increase in MenW IMD in British Columbia started in 2017 and continued to 2020, including causing a localized outbreak in the Okanagan region in late 2017 [37]. In response to this outbreak, the British Columbia Centre for Disease Control was expanding their meningococcal quadrivalent ACWY-conjugate vaccine programme to include individuals 15–19 years of age who usually live, work or attend school/university in the Okanagan region or who planned to travel to the region for 3 weeks or longer and those who have not been vaccinated with a meningococcal quadrivalent vaccine (Frasher Health New Release 18 December 2017). In Alberta, the increase in MenW IMD started in 2018 and lasted until 2022. Both British Columbia and Alberta have quadrivalent meningococcal ACWY-conjugate vaccine booster doses given to grade nine high school children [27].

In Saskatchewan, although the overall percentage of MenW as a cause of IMD was 47.1%, no sustained activity was noticed with most years reporting one or two MenW IMD cases except in 2020 with three cases reported. In Manitoba, the overall percentage of MenW as a cause of IMD was higher at 57.1%, with most years reporting one to three cases except 2023 when ten cases were noted. Data from 2024 appeared to show that there has been a sustained increase in MenW IMD in Manitoba, with 27 cases reported between the period of 21 October 2023 to 18 October 2024 out of a total of 29 IMD cases [38]. In both Ontario and Quebec, the overall percentages of MenW as a cause of IMD were lower at 25.3 and 10.7%, respectively. In Ontario, the proportion of MenW causing IMD was somewhat elevated in 2018, 2019, 2021 and 2022 ranging from 34.4% in 2019 to 45.5% in 2021. In Quebec, an increase in the proportion of MenW in causing IMD was only noticed in 2021 when 37.5% of IMD was due to MenW. There has not been any increase in MenW IMD in the Atlantic provinces. The increase in MenW IMD in Canada was first noticed in Ontario in May 2014 due to a clonal replacement of the endemic MenW ST-22 CC with the known hypervirulent MenW ST-11 CC [39]. Increases in MenW IMD have been reported in several other countries, also due to the same phenomenon of clonal replacement of the endemic clone and expansion of the emerging MenW ST-11 CC [40, 41, 42]. The spread of this so-called South American strain or its variant has occurred globally as well as in Canada [8].

In contrast to this regional increase in IMD due to the MenW strain in Western Canada and the prairie provinces, in Eastern Canada (Quebec and the Atlantic provinces), MenB appeared to be the dominant serogroup causing IMD there. The main clone circulating in Quebec was the ST-269 CC, and in particular strain typed as ST-269. This strain first emerged in Quebec in 2003 [16] and expanded as well as persisted to cause a prolonged increase in MenB IMD [12]. Between 2006 and 2013, the incidence rate of IMD reached 3.4 per 100 000 population in the Saguenay-Lac-Sainte-Jean region, prompting the province to introduce a limited target vaccination with the 4CMenB (Bexsero^®^) vaccine [43,44]. In contrast to the ST-269 strain in Quebec, ST-1161 (a triple locus variant of ST-269 which differs in its *aroE*, *fumC* and *gdh* gene loci) was the only ST-269 CC strain found in Newfoundland and Labrador (one of the Atlantic provinces). In Atlantic Canada between 2009 and 2013, MenB was the dominant serogroup (82.5%) causing IMD, with 73.0% of the MenB belonging to ST-41/44 CC, and in particular a strain characterized as ST-154 [45]. In a follow-up study covering the period 2014–2020, MenB remained as the major (75.0%) serogroup causing IMD, with the ST-154 strain still persisting, whilst other MenB strains had also emerged including ST-269 CC and ST-60 CC [10]. In this study, the MenB strain of ST-154 persisted in Atlantic Canada and recently caused a cluster of cases in Halifax, Nova Scotia, among college students [46]. This outbreak has led to the introduction of a meningococcal B vaccine programme in the province to those residents 25 years and younger attending post-secondary studies and residing in the dormitory or university residence as well as military recruits residing in a congregated living setting such as military barrack [47, 48]. A National Advisory Committee on Immunization has also updated an immunization recommendation for populations at high risk of IMD, including university students [49]. The characteristics of MenB causing prolonged outbreaks, which can span over 10 years, have also been reported elsewhere globally as summarized by [50].

In the province of Quebec, the percentage of IMD due to MenB has decreased yearly over this study period from 84.0% in 2015 to 25.0% in 2023. Concomitant to the decrease in the percentage of MenB, an increase in the percentage of IMD due to MenY has increased year after year, from 8.0% in 2015 to 55.0% in 2023 (Table S3). Unlike the increase in MenY IMD in the USA due to a novel strain typed as ST-1466 (member of the ST-174 CC), the increase in MenY IMD in Quebec was due to a sub-clade of the lineage 23.1 (ST-23 CC), which caused severe disease including meningitis in young adults [11]. The US ST-1466 strain appeared to cause IMD in those between the ages of 30 and 60 years and affected more African Americans as well as people living with HIV. The clinical presentation is also mostly bacteraemia (64%) and septic arthritis. Of those with known outcomes, 18% died, which is higher than the case fatality rate of 11% for MenY IMD in 2017–2021 [51]. In the past 9 years (2015–2023), only six cases of MenY IMD were due to ST-1466, and although half (three cases) happened in 2023 (two in Ontario and one in Quebec), preliminary data from 2024 did not suggest an expansion of this strain in Canada.

Invasive MenC strains in the post-MenC conjugate vaccine era appeared to be somewhat genetically diverse. However, regional differences were noted with the ST-269 CC isolates recovered from the western province of Alberta, whilst the ST-35 CC isolates were recovered from the central or eastern provinces of either Ontario or Quebec, respectively. This is in contrast to the pre-vaccine era from 1999 to 2003 when 96.6% (426 of 441 individual invasive MenC case isolates) were typed as ST-11 CC [52]. Genetic diversification of MenC has also been described after the introduction of the polysaccharide-conjugate vaccines against serogroup C [53].

This study focused on the meningococcal strain types, and as such, we did not collect additional clinical data; nevertheless, using the information provided on the specimen requisition forms has provided us with some interesting facts about IMD in Canada. Firstly, it appears that MenB IMD in general affects a younger age group (median age 20.0 years) when compared to IMD caused by MenC, MenY or MenW (median ages of 48.0, 52.0 and 48.0 years, respectively). This was likely because most MenB cases were caused by the hyper-invasive clonal types such as ST-213 CC, ST-32 CC and ST-269 CC or hypervirulent genotypes like ST-154. When the median ages of MenB cases caused by the ST-213 CC, ST-32 CC, ST-269 CC and ST-154 are examined, their median ages were 25.0, 22.0, 21.0 and 18.0 years, respectively (data not shown). This age difference between MenB cases versus MenC, MenY and MenW cases may potentially be related to the use of the quadrivalent meningococcal conjugate vaccine against serogroups A, C, W and Y in the adolescent booster doses in most provinces and territories in addition to the use of the quadrivalent meningococcal conjugate vaccine as the primary and booster doses in a few provinces and territories.

Also, of the four major serogroups causing IMD in Canada, significantly more MenB (92 out of 292 or 31.5%) and MenC (10 out of 46 or 21.7%) cases appeared to have isolates recovered from the CSF or brain than MenY (23 out of 188 or 12.2%) or MenW (16 out of 232 or 6.9%). The association of capsular groups of meningococci and clinical presentation has also been reported in a study in England with meningitis being more prevalent among MenB cases (28%) [54]. In contrast, a greater percentage of MenC cases (5 out of 46 or 10.9%) appear to have isolates recovered from joints than MenY (6 out of 188 or 3.2%), or MenW (4 out of 232 or 1.7%) or MenB (4 out of 292 or 1.4%). Our findings involving Canadian IMD cases are quite different from those reported in a study of meningococcal septic arthritis (MSA) in England and Wales between the years 2010 and 2020. In that study, of 8081 cases of IMD, 162 cases of MSA were found, 40.7% were due to MenW, 26.5% were due to MenB, 20.4% were due to MenY, 11.7% were due to MenC and 0.6% were due to MenE; despite that, only 16% of all IMD were due to MenW, whilst the majority (67.0%) of all IMD were due to MenB [55]. Our findings are also different from a report in France examining a series of seven arthritis cases secondary to IMD, in which three were due to MenB, three were due to MenC and only one case was due to MenW [56]. What causes these apparent differences is not clear. However, clinical data was not specifically captured in this study, and it is possible that some septic arthritis cases might have been missed in our study.

Although testing requisition forms that are sent to the NML along with the isolates provided demographic information such as age and sex of the case, our study is limited by the fact that outcomes for these patients were not available. It would be beneficial to have this data in order to examine possible correlations between mortality and different meningococcal genetic types. Additionally, case vaccination status is also usually not reported but could provide more insight into how current vaccination programmes are meeting the needs of the Canadian population. Our passive surveillance system also relies on positive reports from the provinces informing us of symptomatic cases; infections that were not recognized as caused by *N. meningitidis* or those that did not present to the health care system (though quite rare) would not be captured under our current strategy.

In conclusion, our study reveals a significant increase in the proportion of invasive meningococcal disease caused by MenW in Canada, most notably in the Western provinces. Though the overall prevalence of MenB has decreased, it remains the most common serogroup causing invasive disease in Central and Eastern Canada, except in the province of Quebec where MenY has emerged to cause a significant percentage (57.7%) of IMD cases in the first 8 months of 2023 [11]. Compared to infections caused by other serogroups, MenB caused IMD in a significantly younger population and is much more clonally diverse. The CNS-associated disease was also more common for MenB and MenC IMD than for other serogroups, which were more likely to involve bacteraemia. This study shows how the distribution and characteristics of IMD in Canada have evolved over the last several years, which can be used to inform public health strategies and vaccination programmes aimed at reducing the burden of IMD.

## Supplementary material

10.1099/jmm.0.001979 Uncited Supplementary Material 1.

## Abbreviations

BIGSdb: bacterial isolate genome sequence database
CC: clonal complex
CNS: central nervous system
CSF: cerebrospinal fluid
IMD: invasive meningococcal disease
MSA: meningococcal septic arthritis
NML: National Microbiology Laboratory
NMLB: National Microbiology Laboratory Branch
ST: sequence type
WGS: whole-genome sequencing
