# Supplementary material for: Geographical and temporal variations of serogroups and clonal types of Neisseria meningitidis involved in culture-confirmed invasive meningococcal disease in Canada, 2015–2023
Source: J Med Microbiol. 2025 Mar 12;74(3):001979. doi: 10.1099/jmm.0.001979 (PMC12282276; doi:10.1099/jmm.0.001979)

Supplementary Table S1.

Temporal distribution of serogroups of *Neisseria meningitidis* from culture-confirmed invasive meningococcal disease cases in Canada, 2015-2023.

Year of isolation	Number (%[+]) of case isolates according to serogroups[*] MenB	MenC	MenY	MenW	Other serogroups[#]	Total
2015	50 (57.5%)	2 (2.3%)	25 (28.7%)	8 (9.2%)	2[a] (2.3%)	87
2016	39 (48.2%)	3 (3.7%)	21 (25.9%)	15 (18.5%)	3[b] (3.7%)	81
2017	37 (38.1%)	8 (8.3%)	25 (25.8%)	27 (27.8%)	0 (0%)	97
2018	32 (27.8%)	5 (4.4%)	25 (21.7%)	51 (44.4%)	2[c] (1.7%)	115
2019	33 (28.2%)	6 (5.1%)	29 (24.8%)	46 (39.3%)	3[d] (2.6%)	117
2020	26 (35.6%)	1 (1.4%)	18 (24.7%)	28 (38.4%)	0 (0%)	73
2021	19 (48.7%)	0 (0%)	2 (5.1%)	18 (46.2%)	0 (0%)	39
2022	26 (40.0%)	8 (12.3%)	12 (18.5%)	19 (29.2%)	0 (0%)	65
2023	30 (30.0%)	13 (13.0%)	31 (31.0%)	20 (20.0%)	6[e] (6.0%)	100
2015-2023	292 (37.7%)	46 (5.9%)	188 (24.3%)	232 (30.0%)	16[f] (2.1%)	774

[+] Percent of total number of isolates in that particular year.

[*] Serogroups: MenB, serogroup B; MenC, serogroup C; MenY, serogroup Y; MenW, serogroup W.

[#] Other serogroups include serogroup E; serogroup Z, and non-encapsulated (NE).

[a] One each of serogroup Z and NE.

[b] Two serogroup E, and one NE.

[c] One each of serogroup E and NE.

[d] Three NE.

[e] One serogroup Z and five NE.

[f] Two serogroup Z, three serogroup E, and eleven NE.

Supplementary Table S2.

Geographical and Temporal variations of invasive meningococcal serogroups in Canada, 2015-2023.

Provinces[¥]/Territories	MenB	MenC	MenY	MenW	Others[+]	Total
BC	23	4	24[*]	64	2 (2 NE)	117
AB	14	6	9	46	1 (E)	76
SK	4	2	3	8	0	17
MB	15	1	4	28	1 (NE)	49
SK/MB	19	3	7	36	1 (NE)	66
ON	79	24	61	58	7 (2E, 1Z, 4 NE)	230
QC	103	8	77	23	5 (1Z, 4NE)	216
NB	10	1	2	1	0	14
NS	27	0	7	3	0	37
NFLD	16	0	0	0	0	16
Atlantic provinces	53	1	10[@]	4	0	68
Territories[#]	1	0	0	1	0	2
Canada	292	46	188	232	16	774

[¥] British Columbia (BC), Alberta (AB), Saskatchewan (SK), Manitoba (MB), Ontario (ON), Quebec (QC), New Brunswick (NB), Nova Scotia (NS), and Newfoundland & Labrador (NFLD).

[+]Other serogroups consist of three serogroup E; two serogroup Z, one non-groupable (NG), Twelve non-encapsulated (NE).

[*] Included one MenY which is typed as 2a and ST-11

[#] Territories included one MenB from Northwest Territories and one MenW from Yukon.

[@]Includes one MenY from Prince Edward Island isolated in 2015.

Supplementary Table S3.

Culture confirmed invasive meningococcal disease (IMD) case isolates in British Columbia, Canada, 2015 to 2023.

British Columbia	MenB	MenC	MenY	MenW	Others	Total
2015	5	0	2	2	0	9
2016	2	0	5	2	1 (NE*)	10
2017	2	1	6*	14	0	23
2018	2	0	5	16	0	23
2019	3	0	3	21	0	27
2020	2	0	2	7	0	11
2021	4	0	0	0	0	4
2022	2	0	0	2	0	4
2023	1	3	1	0	1 (NE)	6
2015 to 2023	23	4	24	64	2 (NE)	117

* NE = non-encapsulated

Culture confirmed invasive meningococcal disease (IMD) case isolates in Alberta, Canada, 2015 to 2023.

Alberta	MenB	MenC	MenY	MenW	Others	Total
2015	4	0	2	1	0	7
2016	2	0	2	2	1 (E+)	7
2017	2	2	0	4	0	8
2018	2	3	3	11	0	19
2019	3	1	0	8	0	12
2020	1	0	0	9	0	10
2021	0	0	0	7	0	7
2022	0	0	1	4	0	5
2023	0	0	1	0	0	1
2015 to 2023	14	6	9	46	1 (E)	76

+ serogroup E

Culture confirmed invasive meningococcal disease (IMD) case isolates in Saskatchewan, Canada, 2015 to 2023.

Saskatchewan	MenB	MenC	MenY	MenW	Others	Total
2015	1	0	0	0	0	1
2016	0	0	1	0	0	1
2017	0	2	0	1	0	3
2018	0	0	1	2	0	3
2019	0	0	1	0	0	1
2020	1	0	0	3	0	4
2021	0	0	0	0	0	0
2022	1	0	0	1	0	2
2023	1	0	0	1	0	2
2015 to 2023	4	2	3	8	0	17

Culture confirmed invasive meningococcal disease (IMD) case isolates in Manitoba, Canada, 2015 to 2023.

Manitoba	MenB	MenC	MenY	MenW	Others	Total
2015	2	0	0	1	0	3
2016	4	0	0	3	0	7
2017	1	0	1	1	0	3
2018	1	0	0	2	0	3
2019	1	1	1	3	1 (NE*)	7
2020	0	0	0	3	0	3
2021	2	0	0	3	0	5
2022	1	0	2	2	0	5
2023	3	0	0	10	0	13
2015 to 2023	15	1	4	28	1 (NE)	49

* NE = non-encapsulated

Culture confirmed invasive meningococcal disease (IMD) case isolates in Ontario, Canada, 2015 to 2023.

Ontario	MenB	MenC	MenY	MenW	Others	Total
2015	10	2	14	3	1 (Z#)	30
2016	7	3	8	5	1 (E+)	24
2017	11	2	9	4	0	26
2018	10	1	8	11	1 (E)	31
2019	8	2	9	11	2 (2 NE*)	32
2020	10	1	7	3	0	21
2021	6	0	0	5	0	11
2022	8	7	0	8	0	23
2023	9	6	6	8	2 (2 NE)	31
2015 to 2023	79	24	61	58	7 (2E, 1Z, 4NE)	229

# serogroup Z
+ serogroup E
* NE = non-encapsulated

Culture confirmed invasive meningococcal disease (IMD) case isolates in Quebec, Canada, 2015 to 2023.

Quebec	MenB	MenC	MenY	MenW	Others	Total
2015	21	0	2	1	1 (NE*)	25
2016	17	0	4	3	0	24
2017	15	1	8	3	0	27
2018	9	1	7	7	1 (NE)	25
2019	12	1	14	0	0	27
2020	9	0	9	3	0	21
2021	3	0	2	3	0	8
2022	7	1	9	2	0	19
2023	10	4	22	1	3 (2NE/1Z#)	40
2015 to 2023	103	8	77	23	5 (4NE, 1Z)	216

* NE = non-encapsulated
# serogroup Z

Culture confirmed invasive meningococcal disease (IMD) case isolates in New Brunswick, Canada, 2015 to 2023.

New Brunswick	MenB	MenC	MenY	MenW	Others	Total
2015	3	0	0	0	0	3
2016	0	0	0	0	0	0
2017	1	0	0	0	0	1
2018	3	0	1	0	0	4
2019	1	1	0	1	0	3
2020	0	0	0	0	0	0
2021	1	0	0	0	0	1
2022	1	0	0	0	0	1
2023	0	0	1	0	0	1
2015 to 2023	10	1	2	1	0	14

Culture confirmed invasive meningococcal disease (IMD) case isolates in Nova Scotia, Canada, 2015 to 2023.

Nova Scotia	MenB	MenC	MenY	MenW	Others	Total
2015	4	0	4	0	0	8
2016	2	0	1	0	0	3
2017	3	0	1	0	0	4
2018	2	0	0	1	0	3
2019	3	0	1	2	0	6
2020	2	0	0	0	0	2
2021	2	0	0	0	0	2
2022	4	0	0	0	0	4
2023	5	0	0	0	0	5
2015 to 2023	27	0	7	3	0	37

Culture confirmed invasive meningococcal disease (IMD) case isolates in Newfoundland and Labrador, Canada, 2015 to 2023.

Newfoundland and Labrador	MenB	MenC	MenY	MenW	Others	Total
2015	0	0	0	0	0	0
2016	4	0	0	0	0	4
2017	2	0	0	0	0	2
2018	3	0	0	0	0	3
2019	2	0	0	0	0	2
2020	1	0	0	0	0	1
2021	1	0	0	0	0	1
2022	2	0	0	0	0	2
2023	1	0	0	0	0	1
2015 to 2023	16	0	0	0	0	16

Other IMD cases not captured in the tables above include:
One MenY in Prince Edward Island (PE) in 2015
One MenB in Northwest Territories (NT) in 2016
One MenW in Yukon (YK) in 2018

Supplementary Table S4A.

Clonal analysis of culture-confirmed invasive serogroup B *Neisseria meningitidis* (MenB) in Canada, 2015-2023.

Number of invasive MenB according to Clonal Complex (CC) by MLST[#]

Province and Territories[*]	ST-41/44 CC[§]	ST-213 CC[¶]	ST-32 CC[¥]	ST-269 CC[∞]	ST-35 CC[π]	ST-60 CC[Ψ]	Other CCs	Unassigned[+]	Total
BC	9	2	6	1	1	0	1[a]	3	23
AB	6	4	1	2	0	0	1[b]	0	14
SK	1	1	0	0	1	0	1[c]	0	4
MB	14	0	0	1	0	0	0	0	15
ON	38	8	5	8	1	0	9[d]	10	79
QC	24	3	7	48	3	1	10[e]	7	103
NB	7	1	0	2	0	0	0	0	10
NS	19	0	1	2	0	0	3[f]	2	27
NFLD	1	0	1	7	0	7	0	0	16
NWT	1	0	0	0	0	0	0	0	1
Canada	120	19	21	71	6	8	25	22	292

[#] MLST = Multi-Locus Sequence Typing

[*]Provinces and Territories: British Columbia (BC), Alberta (AB), Saskatchewan (SK), Manitoba (MB), Ontario (ON), Quebec (QC), New Brunswick (NB), Nova Scotia (NS), Newfoundland and Labrador (NFLD), Northwest Territories (NWT).

[§] Consisted of 36 different STs: 51 isolates of ST-154; 12 isolates of ST-6617; 8 isolates of ST-41; 6 isolates of ST-571; three isolates each of ST-43 and ST-414; two isolates each of ST-44, ST-409, ST-1578, ST-2314, ST-7612, ST-11393, and ST-14915[##]; one isolate each of ST-207, ST-568, ST-839, ST-878, ST-1194, ST-1473, ST-1475, ST-3161, ST-6169, ST6349, ST-6591, ST-6618, ST-6696, ST-11219, ST-11487, ST-11863, ST-13551, ST14368, ST-14905[##], ST-15415, ST-15612, ST-15614, and ST-15756.

[¶] Consisted of six different STs: fourteen isolates of ST-213; one isolate each of ST-3844, ST-4513, ST-9413, ST-13117, and ST-13868.

[¥] Consisted of ten different STs: five isolates of ST-32; three isolates each of ST-7460 and ST-15418; two isolates each of ST-33, ST-6544, and ST-17120[##]; one isolate each of ST-2726, ST-7783, ST-9989, and ST-17483.

[∞] Consisted of 14 different STs: 44 isolates of ST-269; ten isolates of ST-1161; three isolates each of ST-5494 and ST-8924; two isolates of ST-4133; and one isolate each of ST-13, ST-275, ST-283, ST-565, ST-1049, ST-1195, ST-7939, ST-10864, and ST-11386.

[π] Consisted of five different STs: two isolates of ST-35; one isolate each of ST-1417, ST-3626, ST-11827, and ST-12966.

[Ψ] Consisted of three different STs: six isolates of ST-11011; one isolate each of ST-60 and ST-17246[##].

[a] One isolate of ST-1157 (ST-1157 CC)

[b] One isolate of ST-11 (ST-11 CC)
[c] One isolate of ST-1767 (ST-254 CC)
[d] Two isolates of ST-3327 and one isolate of ST-13446 (both ST-865 CC); one isolate of ST-461 (ST-461 CC); one isolate of ST-162 (ST-162 CC); one isolate of ST-1157 (ST-1157 CC); one isolate of ST-12712 (ST-1572 CC); one isolate of ST-12968 (ST-2057 CC); and one isolate of ST-12063 (ST-23 CC)
[e] four isolates of ST-1157 (ST-1157 CC); two isolates of ST-461 (ST-461 CC); one isolate each of ST-162 and ST-10323 (both ST-162 CC); one isolate of ST-103 (ST-103 CC); and one isolate of ST-917 (ST-37 CC)
[f] Two isolates of ST-1157 and one isolate of ST-13682 (both ST-1157 CC)
[+] Unassigned to any known CC: One isolate each of ST-3482, ST-14916[##], and ST-17245[##] from BC; four isolates of ST-5571, and one isolate each of ST-336, ST-2003, ST-6122, ST-11118, ST-15613, ST-17569[##] from ON; two isolates of ST-14831[##], two isolates of ST-938, one isolate each of ST-13456, ST-15200, and one isolate of ST-17906 from QC; two isolates of ST-5571 from NS
[##] Eight novel STs (highlighted), not found previously and new to this study

Supplementary Table S4B.

Clonal analysis of invasive serogroup C *Neisseria meningitidis* (MenC) in Canada, 2015 to 2023.

Number of invasive MenC according to Clonal Complex (CC) by MLST[#]

Provinces[*]	ST-11 CC[§]	ST-269 CC[¶]	ST-35 CC[¥]	Other CCs	Unassigned[+]	Total
BC	4	0	0	0	0	4
AB	2	3	0	0	1	6
SK	2	0	0	0	0	2
MB	0	0	0	1[a]	0	1
ON	20	0	1	2[b]	1	24
QC	4	0	2	1[c]	1	8
NB	1	0	0	0	0	1
Canada	33	3	3	4	3	46

[#] MLST = Multi-Locus Sequence Typing

[*] Provinces: British Columbia (BC), Alberta (AB), Saskatchewan (SK), Manitoba (MB), Ontario (ON), Quebec (QC), New Brunswick (NB).

[§] Consisted of 31 isolates of ST-11; and one isolate each of ST-5752, ST-12819.

[¶] 3 isolates of ST-1195 from AB.

[¥] Two isolates of ST-278 and one isolate of ST-12549.

[a] ST-7516 (ST-41/44 CC)

[b] One isolate of ST-2006 (ST-103 CC) and one isolate of ST-1572 (ST-1572 CC)

[c] One isolate of ST-5133 (ST-103 CC)

[+] Unassigned to any known CC: one isolate of ST-1768 (AB); one isolate of ST-5571 (ON), and one isolate of ST-14884 (QC).

Supplementary Table S4C.

Clonal analysis of invasive serogroup Y *Neisseria meningitidis* (MenY) in Canada, 2015 to 2023.

Number of invasive MenY according to Clonal Complex (CC) by MLST[#]

Province[*]	ST-23 CC[+]	ST-167 CC[§]	ST-174 CC[¶]	Other CCs	Unassigned[+]	Total
BC	13	10	0	1[a]	0	24
AB	6	1	0	0	2	8
SK	2	1	0	0	0	3
MB	1	3	0	0	0	4
ON	49	4	4	3[b]	1	61
QC	63	3	2	2[c]	7	77
NB	1	1	0	0	0	2
NS	4	3	0	0	0	7
PEI	0	1	0	0	0	1
Atlantic Canada	5	5	0	0	0	10
Canada	139 (73.9%)	27 (14.4%)	6 (3.2%)	6 (3.2%)	10 (5.3%)	188

[#] MLST = multi-locus sequence typing

[*] Provinces = British Columbia (BC), Alberta (AB), Saskatchewan (SK), Manitoba (MB), Ontario (ON), Quebec (QC), NB (New Brunswick), Nova Scotia (NS), Prince Edward Island (PIE).

[+] Consisted of 83 isolates of ST-23; 28 isolates of ST-1655; 8 isolates of ST-3582; 5 isolates of ST-10732; 5 isolates of ST-10466; 2 isolates of ST-3587; and one isolate each of 8 other STs (ST-183, ST-4183, ST-6800, ST-10880, ST-11604, ST-12163, ST-13454, ST-14319).

[§] Consisted of 13 isolates of ST-1624; 4 isolates of ST-3980; three isolates of ST-2880; 2 isolates of ST-9579; 2 isolates of ST-11847; and one isolate each of ST-3705, ST-12967, ST-11796.

[¶] All six isolates belonged to ST-1466

[a] ST-11 (ST-11 CC)

[b] One ST-269 (ST-269 CC); two ST-1157 (ST-1157 CC)

[c] One ST-16114 (ST-22 CC); one ST-1157 (ST-1157 CC)

[+] Unassigned = unassigned to any known CC: one isolate each of ST-11603 and ST-14272 in AB; one isolate of ST-11603 in ON; three isolates of ST-6464; three isolates of ST-10908; and one isolate of ST-12965 in QC.

Supplementary Table S4D.

Clonal analysis if invasive serogroup W *Neisseria meningitidis* (MenW) in Canada, 2015 to 2023.

Number of invasive MenW according to Clonal Complex (CC) by MLST[#]

Provinces and Territories[+]	ST-11 CC[*]	ST-22 CC[§]	Other CCs	Total
BC	60	4	0	64
AB	45	1	0	46
SK	7	0	1[a]	8
MB	26	2	0	28
ON	57	1	0	58
QC	18	3	2[b]	23
NB	1	0	0	1
NS	3	0	0	3
YK	1	0	0	1
Canada	218	11	3	232

[#]MLST = Multi-Locus Sequence Typing

[+] Provinces and Territories: British Columbia (BC), Alberta (AB), Saskatchewan (SK), Manitoba (MB), Ontario (ON), Quebec (QC), New Brunswick (NB), Nova Scotia (NS), Yukon (YK).

[*] Consisted of 208 isolates of ST-11; ten isolates of other STs (two isolates of ST-4677; one isolate each of ST-247, ST-3035, ST-3505, ST-11371, ST-12818, ST-13250, ST-14735, ST-14817).

[§] Consisted of five isolates of ST184, and one isolate each of ST-22, ST-1158, ST-1224, ST-1476, ST-8974, ST-15903.

[a] One isolate of ST-1308 (unassigned to any known CC).

[b] One isolate of ST-11739 (ST-60 CC) and one isolate of ST-15377 (ST-9316 CC).

Supplementary Table S5.

Indivdiual line listing of the antigenic and sequence type characteristics of culture-confirmed IMD isolates submitted between 2015 and 2023 in Canada.

Isolate number	Antigenic formula	PorA VR1	PorA VR2	PorA VR3	Sequence Type	Clonal Complex
1	B:1,19:P1.-	18	25-92	38-1	414	ST-41/44 complex/Lineage 3
2	B:1,19:P1.-	22	13-1	35-1	32	ST-32 complex
3	B:1,19:P1.-	18-1	34	38	409	ST-41/44 Complex
4	B:1,19:P1.-	17	16-3	36	213	ST-213 complex
5	B:1,19:P1.-	17	9	35-1	6617	ST-41/44 complex
6	B:1,19:P1.-	22	9	35-1	1161	ST-269 complex
7	B:1,19:P1.-	21	16-36	37-1	new ST	unassigned
8	B:1,19:P1.14	22	14	36	213	ST-213 Complex
9	B:1,19:P1.14	22	14	36	213	ST-213 Complex
10	B:1,19:P1.14	22	14	36	3844	ST-213 Complex
11	B:1,19:P1.14	22	14	36	213	ST-213 Complex
12	B:1,19:P1.14	22	14	36	213	ST-213 complex
13	B:1,19:P1.14	22	14	36	213	ST-213 Complex
14	B:1,19:P1.14	22	14	36	213	ST-213 complex
15	B:1,19:P1.14	22	14	36	9413	ST-213 complex
16	B:1,19:P1.14	22	14	36	213	ST-213 complex
17	B:1,19:P1.14	22	14	36	213	ST-213 complex
18	B:1,19:P1.14	22	14	36	4531	ST-213 complex
19	B:1,19:P1.14	22	14	36	213	ST-213 complex
20	B:1,19:P1.19	19-2	13-1	36	461	ST-461 Complex
21	B:1,19:P1.19	19	13-32	36	461	ST-461 Complex
22	B:1,19:P1.19	19	13-45	36	461	ST-461 complex
23	B:1,19:P1.4	7-2	4	37	6169	ST-41/44 complex
24	B:1,19:P1.6	18	25	38-1	7612	ST-41/44 complex/Lineage 3
25	B:1,19:P1.6	18	25	38-1	12968	ST-2057 complex
26	B:1,19:P1.6	18	25	38-1	2314	ST-41/44 complex/Lineage 3
27	B:1,19:P1.6	18	25	38-1	414	ST-41/44 complex
28	B:1,19:P1.6	18	25	38-1	2314	ST-41/44 complex
29	B:1,19:P1.6	18-4	25	38-1	409	ST-41/44 complex
30	B:1,19:P1.6	18	25-81	38-1	414	ST-41/44 complex
31	B:1,19:P1.7	7	16-26	Absence	17120	ST-32 complex
32	B:1,19:P1.9	17	9	35-1	6617	ST-41/44 Complex/Lineage 3
33	B:1,19:P1.9	17	9	35-1	6617	ST-41/44 Complex/Lineage 3
34	B:1,19:P1.9	17	9	35-1	6617	ST-41/44 complex/lineage 3
35	B:1,19:P1.9	17	9	35-1	6617	ST-41/44 complex
36	B:1,19:P1.9	17	9	35-1	6617	ST-41/44 complex

37	B:1,19:P1.9	17	9	35-1	6617	ST-41/44 complex
38	B:1,19:P1.9	17	9	35-1	6617	ST-41/44 complex
39	B:1,19:P1.9	17	9	35-1	6617	ST-41/44 complex
40	B:1,19:P1.9	17	9	35-1	6617	ST-41/44 complex
41	B:1,19:P1.9	17	9	Absence	3161	ST-41/44 complex
42	B:1:P1.14	22	14	36	17569	unassigned
43	B:14,19:P1.14	22-1	14	38	1578	ST-41/44 complex/lineage 3
44	B:14,19:P1.14	22	14	36	336	None assigned
45	B:14,19:P1.14	22	14	36	12036	ST-23 complex/Cluster A3
46	B:14,19:P1.14	22	14	36	213	ST-213 Complex
47	B:14,19:P1.14	22-1	14	38	1578	ST-41/44 complex/lineage 3
48	B:14,19:P1.14	22	14	36	5571	unassigned
49	B:14,19:P1.15,19	19-8	15	36	6696	ST-41/44 complex
50	B:14,19:P1.9	17	9	35-1	1157	ST-1157 complex
51	B:14:P1.4	7-2	4	37	11219	ST-41/44 complex/Lineage 3
52	B:15,19:P1.15,19	19	15-1	36	43	ST-41/44 complex/Lineage 3
53	B:15,P1.7,16	7	16	35	32	ST-32 Complex/ET-5 complex
54	B:15:P1.-	18-1	30-20	38	269	ST-269 complex
55	B:15:P1.7,16	7	16	35	32	ST-32 Complex/ET-5 complex
56	B:15:P1.7,16	7	16	35	32	ST-32 Complex/ET-5 complex
57	B:15:P1.7,16	7	16	35	6544	ST-32 Complex/ET-5 complex
58	B:15:P1.7,16	7	17	35	2726	ST-32 complex
59	B:15:P1.7,16	7	16	35	15418	ST-32 complex
60	B:15:P1.7,16	7	16	35	15418	ST-32 complex
61	B:15:P1.7,16	7	16	35	6544	ST-32 complex
62	B:15:P1.7,16	7	16	35	32	ST-32 complex
63	B:15:P1.7,16	7	16	35	15418	ST-32 complex
64	B:15:P1.9	17	9	35-1	6617	ST-41/44 complex
65	B:17:P1.-	17	16-4	36	568	ST-41/44 complex/Lineage 3
66	B:17:P1.-	18-1	30-20	38	269	ST-269 Complex
67	B:17:P1.-	18-1	30-20	38	269	ST-269 complex
68	B:17:P1.-	18-1	30-27	38	269	ST-269 Complex
69	B:17:P1.-	17	16-49	36	15613	unassigned
70	B:17:P1.-	22	14-6	36-2	14915	ST-41/44 complex
71	B:17:P1.14	22-1	14	36-2	571	ST-41/44 complex/Lineage 3
72	B:17:P1.19	19-1	15-11	36	269	ST-269 complex
73	B:17:P1.19	19-1	15-11	36	269	ST-269 complex
74	B:17:P1.19	19-1	15-11	36	5494	ST-269 complex
75	B:17:P1.19	19-1	15-11	36	269	ST-269 complex
76	B:17:P1.19	19-1	15-11	36	269	ST-269 complex
77	B:17:P1.19	19-1	15-11	36	269	ST-269 complex
78	B:17:P1.19	19-1	15-11	36	269	ST-269 complex
79	B:17:P1.19	19-1	15-11	36	269	ST-269 complex
80	B:17:P1.19	19-1	15-11	36	269	ST-269 complex

81	B:17:P1.19	19-1	15-11	36	11386	ST-269 complex
82	B:17:P1.19	19-1	15-11	36	269	ST-269 Complex
83	B:17:P1.19	19-1	15-11	36	269	ST-269 Complex
84	B:17:P1.19	19-1	15-11	36	269	ST-269 Complex
85	B:17:P1.19	19-1	15-11	36	269	ST-269 Complex
86	B:17:P1.19	19-1	15-11	36	269	ST-269 complex
87	B:17:P1.19	19-1	15-11	36	269	ST-269 complex
88	B:17:P1.19	19-1	15-11	36	269	ST-269 complex
89	B:17:P1.19	19-1	15-11	36	269	ST-269 complex
90	B:17:P1.19	19-1	15-11	36	7939	ST-269 Complex
91	B:17:P1.19	19-1	15-11	36	269	ST-269 complex
92	B:17:P1.19	19-1	15-11	36	269	ST-269 complex
93	B:17:P1.19	19-1	15-11	36	269	ST-269 complex
94	B:17:P1.19	19-1	15-11	36	269	ST-269 complex
95	B:17:P1.19	19-1	15-11	36	5494	ST-269 complex
96	B:17:P1.19	19-1	15-11	36	269	ST-269 complex
97	B:17:P1.19	19-1	15-11	36	4133	ST-269 complex
98	B:17:P1.19	19-1	15-11	36	269	ST-269 complex
99	B:17:P1.19	19-1	15-11	36	269	ST-269 complex
100	B:17:P1.19	19-1	15-11	36	4133	ST-269 complex
101	B:17:P1.19	19-1	15-11	36	269	ST-269 complex
102	B:17:P1.19	19-1	15-11	36	269	T-269 complex
103	B:17:P1.19	19-1	15-11	36	269	ST-269 complex
104	B:17:P1.19	19-1	15-11	36	8924	ST-269 complex
105	B:17:P1.19	19-1	15-11	36	5494	ST-269 complex
106	B:17:P1.19	19-1	15-11	36	269	ST-269 complex
107	B:17:P1.19	19-1	15-11	36	269	ST-269 complex
108	B:17:P1.19	19-1	15-11	new	269	ST-269 complex
109	B:17:P1.2	5	2	36-2	1767	ST-254 Complex
110	B:17:P1.7	7	16-26	35	9989	ST-32 complex/ET-5 complex
111	B:17:P1.7	7	30	38	269	ST-269 complex
112	B:17:P1.9	18-7	9	35-1	269	ST-269 complex
113	B:17:P1.9	18-7	9	35-1	269	ST-269 Complex
114	B:17:P1.9	18-7	9	35-1	269	ST-269 Complex
115	B:17:P1.9	22	9	35-1	1195	ST-269 complex
116	B:17:P1.9	22	9	35-1	1161	ST-269 complex
117	B:17:P1.9	22	9	35-1	1161	ST-269 complex
118	B:17:P1.9	18-7	9	35-1	269	ST-269 Complex
119	B:17:P1.9	18-7	9	35-1	269	ST-269 Complex
120	B:17:P1.9	22	9	35-1	6591	ST-41/44 complex/Lineage 3
121	B:17:P1.9	22	9	35-1	1161	ST-269 complex
122	B:17:P1.9	22	9	35-1	1161	ST-269 complex
123	B:17:P1.9	22	9	35-1	1161	ST-269 complex
124	B:17:P1.9	22	9	35-1	1161	ST-269 complex

125	B:17:P1.9	22	9	35-1	1161	ST-269 complex
126	B:17:P1.9	22	9	35-1	1161	ST-269 complex
127	B:17:P1.9	22	9	35-1	14916	unassigned
128	B:17:P1.9	22	9	35-1	275	ST-269 complex
129	B:17:P1.9	18-7	9	35-1	269	ST-269 complex
130	B:17:P1.9	17-1	23	37	60	ST-60 complex
131	B:17:P1.9	22	9	35-1	6122	unassigned
132	B:17:P1.9	18-7	9	35-1	269	ST-269 complex
133	B:17:P1.9	22	9	35-1	1161	ST-269 complex
134	B:17:P1.9	18-7	9	35-1	269	ST-269 complex
135	B:19:P1.-	22	14-6	36-2	1473	ST-41/44 complex/Lineage 3
136	B:19:P1.-	18	25	38-1	7612	ST-41/44 complex/Lineage 3
137	B:19:P1.-	22	14-6	36-2	44	ST-41/44 complex/Lineage 3
138	B:19:P1.-	21	16-36	37-1	13446	ST-865 complex
139	B:19:P1.-	12-6	13-4	35-1	13551	ST-41/44 complex lineage 3
140	B:19:P1.-	7-2	13-1	35-1	283	ST-269 complex
141	B:19:P1.14	22	14	36	213	ST-213 Complex
142	B:19:P1.14	22	14	36	1157	ST-1157 complex
143	B:19:P1.16	21-7	16	37-1	1157	ST-1157 complex
144	B:19:P1.16	21-7	16	37-1	1157	ST-1157 complex
145	B:19:P1.19	19	15-35	36	43	ST-41/44 Complex/Lineage 3
146	B:19:P1.19	19	15-71	36	43	ST-41/44 Complex/Lineage 3
147	B:19:P1.2,5	5-1	2-2	36-2	1157	ST-1157 complex
148	B:19:P1.2,5	5-1	2-2	new	1157	ST-1157 complex
149	B:19:P1.2,5	5-1	2-2	36-2	1157	ST-1157 complex
150	B:19:P1.4	7-2	4	37	571	ST-41/44 complex/Lineage 3
151	B:19:P1.4	7-2	4	37	3482	None Assigned
152	B:19:P1.4	7-2	4	37	11487	ST-41/44 complex
153	B:19:P1.4	7-2	4	36	162	ST-162 complex
154	B:19:P1.4	7-2	4	36	10323	ST-162 complex
155	B:19:P1.9	17	9	35-1	1157	ST-1157 complex
156	B:19:P1.9	18-7	9	35-1	571	ST-41/44 complex/Lineage 3
157	B:19:P1.9	22	9	35-1	6618	ST-41/44 Complex/Lineage 3
158	B:19:P1.9	18-7	9	35-1	571	ST-41/44 complex/Lineage 3
159	B:19:P1.9	17	9	35-1	13682	ST-1157 complex
160	B:19:P1.9	17	9	35-1	6617	ST-41/44 complex
161	B:19:P1.9	18-7	9	35-1	571	ST-41/44 complex
162	B:19:P1.9	22	9	35-1	14831	unassigned
163	B:19:P1.9	18-7	9	35-1	571	ST-41/44 complex
164	B:4:P1.-	7-2	4-15	37	41	ST-41/44 complex/Lineage 3
165	B:4:P1.-	7-2	4-15	37	41	ST-41/44 complex/Lineage 3
166	B:4:P1.-	21	16-5	37-1	44	ST-41/44 complex/Lineage 3
167	B:4:P1.-	22	14-6	36-2	11393	ST-41/44 complex/Lineage 3
168	B:4:P1.-	22	14-6	36-2	207	ST-41/44 Complex/Lineage 3

169	B:4:P1.-	22-28	14-6	36-2	14368	ST-41/44 complex/Lineage 3
170	B:4:P1.-	22	14-6	36-2	14915	ST-41/44 complex
171	B:4:P1.-	7-2	13-1	35-1	3626	ST-35 complex
172	B:4:P1.-	22	14-6	36-2	15612	ST-41/44 complex
173	B:4:P1.-	22	14-6	36-2	15756	ST-41/44 complex
174	B:4:P1.-	5-2	10-4	36-2	41	ST41/44 complex
175	B:4:P1.-	18	25	38-1	new	unassigned
176	B:4:P1.-	7-2	4	37	154	ST-41/44 complex
177	B:4:P1.1	7-4	1	35-1	878	ST-41/44 complex/Lineage 3
178	B:4:P1.12	12-1	13-1	35-1	14905	ST-41/44 complex
179	B:4:P1.14	22-1	14	38	35	ST-35 complex
180	B:4:P1.14	22	14	36	213	ST-213 Complex
181	B:4:P1.14	22-1	14	38	12966	ST-35 complex
182	B:4:P1.14	22	14	36	5571	unassigned
183	B:4:P1.14	22-1	14	38	35	ST-35 complex
184	B:4:P1.14	22-1	14	38	41	ST41/44 complex
185	B:4:P1.14	22	14	36	5571	Unassigned
186	B:4:P1.14	22	14-3	36	13868	ST-213 complex
187	B:4:P1.14	22	14	36	213	ST-213 complex
188	B:4:P1.14	22	14	new	5571	unassigned
189	B:4:P1.15,19	19-1	15	36	7783	ST-32 Complex/ET-5 Complex
190	B:4:P1.15,19	19	15	36	938	unassigned
191	B:4:P1.15,19	19	15-1	36-2	11827	ST-35 Complex
192	B:4:P1.16	21	16	37-1	839	ST-41/44 complex/Lineage 3
193	B:4:P1.16	21	16-36	37-1	3327	ST-865 complex
194	B:4:P1.19	15-1	19-11	36	8924	ST-269 complex
195	B:4:P1.2,5	5	2	36-2	33	ST-32 complex
196	B:4:P1.4	7-2	4	37	154	ST-41/44 complex/Lineage 3
197	B:4:P1.4	7-2	4	37	154	ST-41/44 complex/Lineage 3
198	B:4:P1.4	7-2	4	37	154	ST-41/44 complex/Lineage 3
199	B:4:P1.4	7-2	4	37	154	ST-41/44 complex/Lineage 3
200	B:4:P1.4	7-2	4	37	154	ST-41/44 complex/Lineage 3
201	B:4:P1.4	7-2	4	37	154	ST-41/44 complex/Lineage 3
202	B:4:P1.4	7-2	4	37	154	ST-41/44 complex/Lineage 3
203	B:4:P1.4	7-2	4	37	154	ST-41/44 complex/Lineage 3
204	B:4:P1.4	7-2	4	37	11863	ST-41/44 complex/Lineage 3
205	B:4:P1.4	7-2	4	37	154	ST-41/44 complex/Lineage 3
206	B:4:P1.4	7-2	4	37	154	ST-41/44 complex/Lineage 3
207	B:4:P1.4	7-2	4	37	154	ST-41/44 complex/Lineage 3
208	B:4:P1.4	7-2	4	37	154	ST-41/44 complex/Lineage 3
209	B:4:P1.4	7-2	4	37	154	ST-41/44 complex/Lineage 3
210	B:4:P1.4	7-2	4	37	154	ST-41/44 complex/Lineage 3
211	B:4:P1.4	7-2	4	37	154	ST-41/44 complex/Lineage 3
212	B:4:P1.4	7-2	4	37	154	ST-41/44 complex/Lineage 3

213	B:4:P1.4	7-2	4	37	154	ST-41/44 complex/Lineage 3
214	B:4:P1.4	7-2	4	37	154	ST-41/44 complex/Lineage 3
215	B:4:P1.4	7-2	4	37	154	ST-41/44 complex/Lineage 3
216	B:4:P1.4	7-2	4	37	154	ST-41/44 complex/Lineage 3
217	B:4:P1.4	7-2	4	37	154	ST-41/44 complex/Lineage 3
218	B:4:P1.4	7-2	4	37	154	ST-41/44 complex/Lineage 3
219	B:4:P1.4	7-2	4	37	154	ST-41/44 complex
220	B:4:P1.4	7-2	4	37	154	ST-41/44 complex
221	B:4:P1.4	7-2	4	37	154	ST-41/44 complex
222	B:4:P1.4	7-2	4	37	154	ST-41/44 complex
223	B:4:P1.4	7-2	4	37	154	ST-41/44 complex
224	B:4:P1.4	7-2	4	37	15415	ST-41/44 complex
225	B:4:P1.4	7-2	4	37	154	ST-41/44 complex
226	B:4:P1.4	7-2	4	37	154	ST-41/44 complex
227	B:4:P1.4	7-2	4	37	15614	ST-41/44 complex
228	B:4:P1.4	7-2	4	37	154	ST-41/44 complex
229	B:4:P1.4	7-2	4	37	154	ST-41/44 complex/Lineage 3
230	B:4:P1.4	7-2	4	37	154	ST-41/44 complex
231	B:4:P1.4	7-2	4	37	154	ST-41/44 complex
232	B:4:P1.4	7-2	4	37	154	ST-41/44 complex
233	B:4:P1.4	7-2	4	37	154	ST-41/44 complex
234	B:4:P1.4	7-2	4	37	154	ST-41/44 complex
235	B:4:P1.4	7-2	4	37	154	ST-41/44 complex
236	B:4:P1.4	7-2	4	37	154	ST-41/44 complex
237	B:4:P1.4	7-2	4	37	154	ST-41/44 complex
238	B:4:P1.4	7-2	4	37	154	ST-41/44 complex
239	B:4:P1.4	7-2	4	37	154	ST-41/44 complex
240	B:4:P1.4	7-2	4	37	154	ST-41/44 complex
241	B:4:P1.4	7-2	4	37	154	ST-41/44 complex
242	B:4:P1.4	7-2	4	37	154	ST-41/44 complex
243	B:4:P1.4	7-2	4	37	154	ST-41/44 complex
244	B:4:P1.4	7-2	4	37	154	ST-41/44 complex
245	B:4:P1.4	7-2	4	37	154	ST-41/44 complex
246	B:4:P1.4	7-2	4	37	154	ST-41/44 complex
247	B:4:P1.4	7-2	4	37	154	ST-41/44 complex
248	B:4:P1.6	18	25	38-1	13456	unassigned
249	B:4:P1.6	18	25	38-1	938	unassigned
250	B:4:P1.6	18	25-75	38-1	41	ST41/44 complex
251	B:4:P1.6	18	25	38-1	41	ST-41/44 complex
252	B:4:P1.7,15	7-12	15	36	12712	ST-1572 complex
253	B:4:P1.7,16	7	16	35	17483	ST-32 complex
254	B:4:P1.9	22	9	35-1	41	ST-41/44 complex/Lineage 3
255	B:4:P1.9	17	9	35-1	2003	unassigned
256	B:NT:P1.-	7-2	13-1	35-1	917	ST-37 complex

257	B:NT:P1.-	22	14-6	36-2	6349	ST-41/44 complex/Lineage 3
258	B:NT:P1.-	22	14-6	36-2	11393	ST-41/44 complex
259	B:NT:P1.-	21-2	16-4	37	15200	unassigned
260	B:NT:P1.-	5-2	10-4	36-2	41	ST-41/44 complex
261	B:NT:P1.-	18-1	30	38	7460	ST-32 complex
262	B:NT:P1.1	new	1	35-1	7460	ST-32 complex
263	B:NT:P1.12,13	12-1	13	35-1	103	ST-103 complex
264	B:NT:P1.13,19	19	13	35-1	565	ST-269 Complex
265	B:NT:P1.14	22	14	36	11118	unassigned
266	B:NT:P1.14	22	14	36	5571	unassigned
267	B:NT:P1.14	18-1	14	36	1194	ST-41/44 complex lineage 3
268	B:NT:P1.14	22	14	36	5571	unassigned
269	B:NT:P1.14	22	14	36	7460	ST-32 complex
270	B:NT:P1.15,19	19	15-1	36	10864	ST-269
271	B:NT:P1.16	31	16	37-1	13	ST-269 complex
272	B:NT:P1.19	19-1	15-11	36	8924	ST-269 complex
273	B:NT:P1.2	5	2	36-2	11011	ST-60 complex
274	B:NT:P1.2	5	2	36-2	11011	ST-60 complex
275	B:NT:P1.2,5	5	2	36-2	11011	ST-60 Complex
276	B:NT:P1.2,5	5	2	36-2	11011	ST-60 Complex
277	B:NT:P1.2,5	5	2	36-2	11011	ST-60 Complex
278	B:NT:P1.2,5	5	2	36-2	33	ST-32 complex
279	B:NT:P1.2,5	5	2	36-2	17246	ST-60 complex
280	B:NT:P1.4	7-2	4	37	1475	ST-41/44 Complex/Lineage 3
281	B:NT:P1.4,7	7-2	4	37	154	ST-41/44 complex/Lineage 3
282	B:NT:P1.4,7	7-2	4	36	162	ST-162 complex
283	B:NT:P1.5	5-1	10-8	36-2	11	ST-11 complex/37 complex
284	B:NT:P1.5	5-3	10-1	36-1	17245	Unassigned
285	B:NT:P1.7	7	30-3	38	1049	ST-269 Complex
286	B:NT:P1.7	7	30	38	11011	ST-60 Complex
287	B:NT:P1.9	18-7	9	35-1	269	ST-269 complex
288	B:NT:P1.9	18-7	9	35-1	269	ST-269 complex
289	B:NT:P1.9	22	9	35-1	1417	ST-35 complex
290	B:NT:P1.9	22	9	35-1	13117	ST-213 complex
291	B:NT:P1.9	22	9	35-1	14831	unassigned
292	B;1,19:P1.7	7	16-26	35	17120	ST-32 complex
293	C:1,19:P1.15,19	19	15	36	14884	unassigned
294	C:15,19:P1.13	7-2	13	35-1	278	ST-35 complex
295	C:15,19:P1.13	7-2	13	35-1	278	ST-35 complex
296	C:15,19:P1.4	21-27	4	37	1768	Unassigned
297	C:2a:P1.-	5	2	36-2	11	ST-11 complex/37 complex
298	C:2a:P1.2	5	2	36-2	11	ST-11 complex/37 complex
299	C:2a:P1.2,5	5	2	36-2	5752	ST-11 complex/37 complex
300	C:2a:P1.2,5	5	2	36-2	12819	ST-11 complex/37 complex

301	C:2a:P1.2,5	5	2	36-2	11	ST-11 complex/37 complex
302	C:2a:P1.2,5	5	2	36-2	11	ST-11 complex/37 complex
303	C:2a:P1.2,5	5	2	36-2	11	ST-11 complex/37 complex
304	C:2a:P1.2,5	5	2	36-2	11	ST-11 complex/37 complex
305	C:2a:P1.2,5	5	2	36-2	11	ST-11 complex/37 complex
306	C:2a:P1.5	5-1	10-8	36-2	11	ST-11 complex/37 complex
307	C:2a:P1.5	5-1	10-8	36-2	11	ST-11 complex/37 complex
308	C:2a:P1.5	5-1	10-8	36-2	11	ST-11 complex/37 complex
309	C:2a:P1.5	5-1	10-8	36-2	11	ST-11 complex/37 complex
310	C:2a:P1.5	5-1	10-8	36-2	11	ST-11 complex/37 complex
311	C:2a:P1.5	5-1	10-8	36-2	11	ST-11 complex
312	C:2a:P1.5	5-1	10-8	36-2	11	ST-11 complex
313	C:2a:P1.5	5-1	10-8	36-2	11	ST-11 complex
314	C:2a:P1.5	5-1	10-8	36-2	11	ST-11 complex
315	C:2a:P1.5	5-1	10-8	36-2	11	ST-11 complex
316	C:2a:P1.5	5-1	10-8	36-2	11	ST-11 complex
317	C:2a:P1.5	5-1	10-8	36-2	11	ST-11 complex
318	C:2a:P1.5	5-1	10-8	36-2	11	ST-11 complex
319	C:2a:P1.5	5-1	10-8	36-2	11	ST-11 complex
320	C:2a:P1.5	5-1	10-8	36-2	11	ST-11 complex
321	C:2a:P1.5	5-1	10-8	36-2	11	ST-11 complex
322	C:2a:P1.5	5-1	10-8	36-2	11	ST-11 complex
323	C:2a:P1.5	5-1	10-8	36-2	11	ST-11 complex
324	C:2a:P1.5	5-1	10-8	36-2	11	ST-11 complex
325	C:2a:P1.5	5-1	10-8	36-2	11	ST-11 complex
326	C:2a:P1.5	5-1	10-8	36-2	11	ST-11 complex
327	C:2a:P1.5	5-1	10-8	36-2	11	ST-11 complex
328	C:4:P1.14	22-1	14	38	12549	ST-35 Complex
329	C:4:P1.14	7-36	14	35-1	1572	ST-1572 complex
330	C:NT:P1.-	18-1	3	38	5133	ST-103 complex
331	C:NT:P1.-	12-6	13-4	35-1	7516	ST-41/44 complex
332	C:NT:P1.-	5-1	10-8	36-2	11	ST-11 complex
333	C:NT:P1.14	22	14	36	5571	unassigned
334	C:NT:P1.5	5-1	10-4	36-2	2006	ST-103 complex
335	C:NT:P1.5	5-1	10-8	36-2	11	ST-11 complex
336	C:NT:P1.9	22	9	35-1	1195	ST-269 complex
337	C:NT:P1.9	22	9	35-1	1195	ST-269 complex
338	C:NT:P1.9	22	9	35-1	1195	ST-269 complex
339	W:2a:P1.-	5	2	36-2	12818	ST-11 complex/37 complex
340	W:2a:P1.-	5	2	36-2	11	ST-11 complex/37 complex
341	W:2a:P1.-	5	2	36-2	11	ST-11 complex/37 complex
342	W:2a:P1.-	5	2	36-2	11	ST-11 complex/37 complex
343	W:2a:P1.-	5	2	36-2	11	ST-11 complex
344	W:2a:P1.-	5	2	36-2	14817	ST-11 complex

345	W:2a:P1.-	5	2	36-2	11	ST-11 complex
346	W:2a:P1.-	5	2	36-2	11	ST-11 complex
347	W:2a:P1.-	5	2	36-2	11	ST-11 complex
348	W:2a:P1.-	5	2	36-2	11	ST-11 complex
349	W:2a:P1.2	5	2	36-2	11	ST-11 complex/37 complex
350	W:2a:P1.2	5	2	36-2	11	ST-11 complex/37 complex
351	W:2a:P1.2	5	2	36-2	11	ST-11 complex/37 complex
352	W:2a:P1.2	5	2	36-2	11	ST-11 complex/37 complex
353	W:2a:P1.2	5	2	36-2	13250	ST-11 complex/37 complex
354	W:2a:P1.2	5	2	36-2	11	ST-11 complex/37 complex
355	W:2a:P1.2	5	2	36-2	11	ST-11 complex/37 complex
356	W:2a:P1.2	5	2	36-2	11	ST-11 complex/37 complex
357	W:2a:P1.2	5	2	36-2	11	ST-11 complex/37 complex
358	W:2a:P1.2	5	2	36-2	11	ST-11 complex/37 complex
359	W:2a:P1.2	5	2	36-2	11	ST-11 complex/37 complex
360	W:2a:P1.2	5	2	36-2	11	ST-11 complex/37 complex
361	W:2a:P1.2	5	2	36-2	11	ST-11 complex/37 complex
362	W:2a:P1.2	5	2	36-2	11	ST-11 complex/37 complex
363	W:2a:P1.2	5	2	36-2	11	ST-11 complex/37 complex
364	W:2a:P1.2	5	2	36-2	11	ST-11 complex/37 complex
365	W:2a:P1.2	5	2	36-2	11	ST-11 complex/37 complex
366	W:2a:P1.2	new	2	36-2	11	ST-11 complex/37 complex
367	W:2a:P1.2	5	2	36-2	11	ST-11 complex/37 complex
368	W:2a:P1.2	5	2	36-2	11	ST-11 complex/37 complex
369	W:2a:P1.2	5	2	36-2	14735	ST-11 complex/37 complex
370	W:2a:P1.2	5	2	36-2	11	ST-11 complex/37 complex
371	W:2a:P1.2	5	2	36-2	11	ST-11 complex/37 complex
372	W:2a:P1.2	5	2	36-2	11	ST-11 complex/37 complex
373	W:2a:P1.2	5	2	36-2	11	ST-11 complex/37 complex
374	W:2a:P1.2	5	2	36-2	11	ST-11 complex/37 complex
375	W:2a:P1.2	5	2	36-2	11	ST-11 complex/37 complex
376	W:2a:P1.2	5	2	36-2	11	ST-11 complex/37 complex
377	W:2a:P1.2	5	2	36-2	11	ST-11 complex/37 complex
378	W:2a:P1.2	5	2	36-2	4677	ST-11 complex/37 complex
379	W:2a:P1.2	5	2	36-2	11	ST-11 complex/37 complex
380	W:2a:P1.2	5	2	36-2	11	ST-11 complex/37 complex
381	W:2a:P1.2	5	2	36-2	11	ST-11 complex/37 complex
382	W:2a:P1.2	5	2	36-2	11	ST-11 complex/37 complex
383	W:2a:P1.2	5	2	36-2	11	ST-11 complex/37 complex
384	W:2a:P1.2	5	2	36-2	11	ST-11 complex/37 complex
385	W:2a:P1.2	5	2	36-2	11	ST-11 complex/37 complex
386	W:2a:P1.2	5	2	36-2	11	ST-11 complex/37 complex
387	W:2a:P1.2	5	2	36-2	11	ST-11 complex/37 complex
388	W:2a:P1.2	5	2	36-2	11	ST-11 complex/37 complex

389	W:2a:P1.2	5	2	36-2	11	ST-11 complex/37 complex
390	W:2a:P1.2	5	2	36-2	11	ST-11 complex/37 complex
391	W:2a:P1.2	5	2	36-2	11	ST-11 complex
392	W:2a:P1.2	5	2	36-2	11	ST-11 complex
393	W:2a:P1.2	5	2	36-2	11	ST-11 complex/37 complex
394	W:2a:P1.2	5	2	36-2	3505	ST-11 complex
395	W:2a:P1.2	5	2	36-2	11	ST-11 complex
396	W:2a:P1.2	5	2	36-2	11	ST-11 complex
397	W:2a:P1.2	5	2	36-2	11	ST-11 complex
398	W:2a:P1.2	5	2	36-2	11	ST-11 complex
399	W:2a:P1.2	5	2	36-2	11	ST-11 complex
400	W:2a:P1.2	5	2	36-2	11	ST-11 complex
401	W:2a:P1.2	5	2	36-2	11	ST-11 complex
402	W:2a:P1.2	5	2	36-2	11	ST-11 complex
403	W:2a:P1.2	5	2	36-2	11	ST-11 complex
404	W:2a:P1.2	5	2	36-1	11	ST-11 complex
405	W:2a:P1.2	5	2	36-2	11	ST-11 complex
406	W:2a:P1.2	5	2	36-2	11	ST-11 complex
407	W:2a:P1.2	5	2	36-2	11	ST-11 complex
408	W:2a:P1.2	5	2	36-2	11	ST-11 complex
409	W:2a:P1.2	5	2	36-2	11	ST-11 complex
410	W:2a:P1.2	5	2	36-2	11	ST-11 complex
411	W:2a:P1.2	5	2	36-2	11	ST-11 complex
412	W:2a:P1.2	5	2	36-2	11	ST-11 complex
413	W:2a:P1.2	5	2	36-2	11	ST-11 complex
414	W:2a:P1.2	5	2	36-2	11	ST-11 complex
415	W:2a:P1.2	5	2	36-2	11	ST-11 complex
416	W:2a:P1.2	5	2	36-2	11	ST-11 complex
417	W:2a:P1.2	5	2	36-2	11	ST-11 complex
418	W:2a:P1.2	5	2	36-2	11	ST-11 complex
419	W:2a:P1.2	5	2	36-2	11	ST-11 complex
420	W:2a:P1.2,5	5	2	36-2	11	ST-11 complex/37 complex
421	W:2a:P1.2,5	5	2	36-2	11	ST-11 complex/37 complex
422	W:2a:P1.2,5	5	2	36-2	11	ST-11 complex/37 complex
423	W:2a:P1.2,5	5	2	36-2	11	ST-11 complex/37 complex
424	W:2a:P1.2,5	5	2	36-2	11	ST-11 complex/37 complex
425	W:2a:P1.2,5	5	2	36-2	11	ST-11 complex/37 complex
426	W:2a:P1.2,5	5	2	36-2	11	ST-11 complex/37 complex
427	W:2a:P1.2,5	5	2	36-2	11	ST-11 complex/37 complex
428	W:2a:P1.2,5	5	2	36-2	11	ST-11 complex/37 complex
429	W:2a:P1.2,5	5	2	36-2	11	ST-11 complex/37 complex
430	W:2a:P1.2,5	5	2	36-2	11	ST-11 complex/37 complex
431	W:2a:P1.2,5	5	2	36-2	11	ST-11 complex/37 complex
432	W:2a:P1.2,5	5	2	36-2	11	ST-11 complex/37 complex

433	W:2a:P1.2,5	5	2	36-2	11	ST-11 complex/37 complex
434	W:2a:P1.2,5	5	2	36-2	11	ST-11 complex/37 complex
435	W:2a:P1.2,5	5	2	36-2	11	ST-11 complex/37 complex
436	W:2a:P1.2,5	5	2	36-2	11	ST-11 complex/37 complex
437	W:2a:P1.2,5	5	2	36-2	11	ST-11 complex/37 complex
438	W:2a:P1.2,5	5	2	36-2	11	ST-11 complex/37 complex
439	W:2a:P1.2,5	5	2	36-2	11	ST-11 complex/37 complex
440	W:2a:P1.2,5	5	2	36-2	11	ST-11 complex/37 complex
441	W:2a:P1.2,5	5	2	36-2	11	ST-11 complex/37 complex
442	W:2a:P1.2,5	5	2	36-2	11	ST-11 complex/37 complex
443	W:2a:P1.2,5	5	2	36-2	11	ST-11 complex/37 complex
444	W:2a:P1.2,5	5	2	36-2	11	ST-11 complex/37 complex
445	W:2a:P1.2,5	5	2	36-2	11	ST-11 complex/37 complex
446	W:2a:P1.2,5	5	2	36-2	11	ST-11 complex/37 complex
447	W:2a:P1.2,5	5	2	36-2	11	ST-11 complex/37 complex
448	W:2a:P1.2,5	5	2	36-2	11	ST-11 complex/37 complex
449	W:2a:P1.2,5	5	2	36-2	11	ST-11 complex/37 complex
450	W:2a:P1.2,5	5	2	36-2	11	ST-11 complex/37 complex
451	W:2a:P1.2,5	5	2	36-2	11	ST-11 complex/37 complex
452	W:2a:P1.2,5	5	2	36-2	11739	ST-60 complex
453	W:2a:P1.2,5	5	2	36-2	11	ST-11 complex/37 complex
454	W:2a:P1.2,5	5	2	36-2	11	ST-11 complex/37 complex
455	W:2a:P1.2,5	5	2	36-2	11	ST-11 complex/37 complex
456	W:2a:P1.2,5	5	2	36-2	11	ST-11 complex/37 complex
457	W:2a:P1.2,5	5	2	36-2	11	ST-11 complex/37 complex
458	W:2a:P1.2,5	5	2	36-2	11	ST-11 complex/37 complex
459	W:2a:P1.2,5	5	2	36-2	11	ST-11 complex/37 complex
460	W:2a:P1.2,5	5	2	36-2	11	ST-11 complex/37 complex
461	W:2a:P1.2,5	5	2	36-2	11	ST-11 complex/37 complex
462	W:2a:P1.2,5	5	2	36-2	11	ST-11 complex/37 complex
463	W:2a:P1.2,5	5	2	36-2	11	ST-11 complex/37 complex
464	W:2a:P1.2,5	5	2	36-2	11	ST-11 complex/37 complex
465	W:2a:P1.2,5	5	2	36-2	11	ST-11 complex/37 complex
466	W:2a:P1.2,5	5	2	36-2	11	ST-11 complex/37 complex
467	W:2a:P1.2,5	5	2	36-2	11	ST-11 complex/37 complex
468	W:2a:P1.2,5	5	2	36-2	11	ST-11 complex/37 complex
469	W:2a:P1.2,5	5	2	36-2	11	ST-11 complex/37 complex
470	W:2a:P1.2,5	5	2	36-2	11	ST-11 complex/37 complex
471	W:2a:P1.2,5	5	2	36-2	11	ST-11 complex/37 complex
472	W:2a:P1.2,5	5	2	36-2	11	ST-11 complex/37 complex
473	W:2a:P1.2,5	5	2	36-2	11	ST-11 complex/37 complex
474	W:2a:P1.2,5	5	2	36-2	4677	ST-11 complex
475	W:2a:P1.2,5	5	2	36-2	11	ST-11 complex/37 complex
476	W:2a:P1.2,5	5	2	36-2	11	ST-11 complex

477	W:2a:P1.2,5	5	2	36-2	11	ST-11 complex
478	W:2a:P1.2,5	5	2	36-2	11	ST-11 complex
479	W:2a:P1.2,5	5	2	36-2	11	ST-11 complex
480	W:2a:P1.2,5	5	2	36-2	11	ST-11 complex
481	W:2a:P1.2,5	5	2	36-2	11	ST-11 complex
482	W:2a:P1.2,5	5	2	36-2	11	ST-11 complex
483	W:2a:P1.2,5	5	2	36-2	11	ST-11 complex
484	W:2a:P1.2,5	5	2	36-2	11	ST-11 complex
485	W:2a:P1.2,5	5	2	36-2	11	ST-11 complex
486	W:2a:P1.2,5	5	2	36-2	11	ST-11 complex
487	W:2a:P1.2,5	5	2	36-2	11	ST-11 complex
488	W:2a:P1.2,5	5	2	36-2	11	ST-11 complex
489	W:2a:P1.2,5	5	2	36-2	11	ST-11 complex
490	W:2a:P1.2,5	5	2	36-2	11	ST-11 complex
491	W:2a:P1.2,5	5	2	36-2	11	ST-11 complex
492	W:2a:P1.2,5	5	2	36-2	11	ST-11 complex
493	W:2a:P1.2,5	5	2	36-2	11	ST-11 complex
494	W:2a:P1.2,5	5	2	36-2	11	ST-11 complex
495	W:2a:P1.2,5	5	2	36-2	11	ST-11 complex
496	W:2a:P1.2,5	5	2	36-2	11	ST-11 complex
497	W:2a:P1.2,5	5	2	36-2	11	ST-11 complex
498	W:2a:P1.2,5	5	2	36-2	11	ST-11 complex
499	W:2a:P1.2,5	5	2	36-2	11	ST-11 complex
500	W:2a:P1.2,5	5	2	36-2	11	ST-11 complex
501	W:2a:P1.2,5	5	2	36-2	11	ST-11 complex
502	W:2a:P1.2,5	5	2	36-2	11	ST-11 complex
503	W:2a:P1.2,5	5	2	8	11	ST-11 complex
504	W:2a:P1.2,5	5	2	36-2	11	ST-11 complex
505	W:2a:P1.2,5	5	2	36-2	11	ST-11 complex/37 complex
506	W:2a:P1.2,5	5	2	36-2	11	ST-11 complex
507	W:2a:P1.2,5	5	2	36-2	11	ST-11 complex
508	W:2a:P1.2,5	5	2	36-2	11	ST-11 complex
509	W:2a:P1.2,5	5	2	36-2	11	ST-11 complex
510	W:2a:P1.2,5	5	2	36-2	11	ST-11 complex
511	W:2a:P1.2,5	5	2	36-2	11	ST-11 complex
512	W:2a:P1.2,5	5	2	36-2	11	ST-11 complex
513	W:2a:P1.2,5	5	2	36-2	11	ST-11 complex
514	W:2a:P1.2,5	5	2	36-2	11	ST-11 complex
515	W:2a:P1.2,5	5	2	36-2	11	ST-11 complex
516	W:2a:P1.2,5	5	2	36-2	11	ST-11 complex
517	W:2a:P1.2,5	5	2	36-2	11	ST-11 complex
518	W:2a:P1.2,5	5	2	36-2	11	ST-11 complex
519	W:2a:P1.2,5	5	2	36-2	11	ST-11 complex
520	W:2a:P1.2,5	5	2	36-2	11	ST-11 complex

521	W:2a:P1.2,5	5	2	36-2	11	ST-11 complex
522	W:2a:P1.2,5	5	2	36-2	11	ST-11 complex
523	W:2a:P1.2,5	5	2	36-2	11	ST-11 complex
524	W:2aP1.2,5	5	2	36-2	11	ST-11 complex
525	W:4:P1.16	21	16	37-1	1308	unassigned
526	W:NT:P1.-	5	2	36-2	11	ST-11 complex/37 complex
527	W:NT:P1.-	5	2	36-2	11	ST-11 complex/37 complex
528	W:NT:P1.-	5	2	36-2	11	ST-11 complex/37 complex
529	W:NT:P1.-	5-2	10-49	36-2	15377	ST-9316 complex
530	W:NT:P1.2	5	2	36-2	11	ST-11 complex/37 complex
531	W:NT:P1.2	5	2	36-2	11	ST-11 complex/37 complex
532	W:NT:P1.2	5	2	36-2	11	ST-11 complex/37 complex
533	W:NT:P1.2	5	2	36-2	11	ST-11 complex
534	W:NT:P1.2	5	2	36-2	247	ST-11 complex
535	W:NT:P1.2	5	2	36-2	11	ST-11 complex
536	W:NT:P1.2	5	2	36-2	11	ST-11 complex
537	W:NT:P1.2	5	2	36-2	3035	ST-11 complex
538	W:NT:P1.2	5	2	36-2	11	ST-11 complex
539	W:NT:P1.2	5	2	36-2	11	ST-11 complex
540	W:NT:P1.2,5	5	2	36-2	11	ST-11 complex/37 complex
541	W:NT:P1.2,5	5	2	36-2	11	ST-11 complex/37 complex
542	W:NT:P1.2,5	5	2	36-2	11	ST-11 complex/37 complex
543	W:NT:P1.2,5	5	2	36-2	11	ST-11 complex/37 complex
544	W:NT:P1.2,5	5	2	36-2	11	ST-11 complex/37 complex
545	W:NT:P1.2,5	5	2	36-2	11	ST-11 complex/37 complex
546	W:NT:P1.2,5	5	2	36-2	11	ST-11 complex
547	W:NT:P1.2,5	5	2	36-2	11	ST-11 complex
548	W:NT:P1.2,5	5	2	36-2	11	ST-11 complex
549	W:NT:P1.2,5	5	2	36-2	11	ST-11 complex
550	W:NT:P1.2,5	5	2	36-2	11	ST-11 complex
551	W:NT:P1.2,5	5	2	36-2	11	ST-11 complex
552	W:NT:P1.6	18-1	3	38	184	ST-22 Complex
553	W:NT:P1.6	18-1	3	38	1224	ST-22 complex
554	W:NT:P1.6	18-1	3	38	184	ST-22 Complex
555	W:NT:P1.6	18-1	3	38	1158	ST-22 Complex
556	W:NT:P1.6	18-1	3	38	22	ST-22 Complex
557	W:NT:P1.6	18-1	3	38	8974	ST-22 complex
558	W:NT:P1.6	18-1	3	38	15903	ST-22 complex
559	W:NT:P1.6	18-1	3	38	184	ST-22 complex
560	W135:2a:P1.2	5	2	36-2	11371	ST-11 complex/37 complex
561	W135:2a:P1.2	5	2	36-2	11	ST-11 complex/37 complex
562	W135:2a:P1.2	5	2	36-2	11	ST-11 complex/37 complex
563	W135:2a:P1.2	5	2	36-2	11	ST-11 complex/37 complex
564	W135:2a:P1.2,5	5	2	36-2	11	ST-11 complex/37 complex

565	W135:2a:P1.2,5	5	2	36-2	11	ST-11 complex/37 complex
566	W135:2a:P1.2,5	5	2	36-2	11	ST-11 complex/37 complex
567	W135:2a:P1.2,5	5	2	36-2	11	ST-11 complex/37 complex
568	W135:NT:P1.-	deletion	deletion	deletion	184	ST-22 complex
569	W135:NT:P1.16	18-1	16	37-1	184	ST-22 Complex
570	W135:NT:P1.6	18-1	3	38	1476	ST-22 Complex
571	Y:14,19:P1.	5-2	10-1	36-2	23	ST-23 complex/Cluster A3
572	Y:14,19:P1.	5-2	10-1	36-2	23	ST-23 complex/Cluster A3
573	Y:14,19:P1.-	5-2	10-1	36-2	23	ST-23 complex/Cluster A3
574	Y:14,19:P1.-	5-2	10-1	36-2	23	ST-23 complex/Cluster A3
575	Y:14,19:P1.-	5-2	10-1	36-2	23	ST-23 complex/Cluster A3
576	Y:14,19:P1.-	5-2	10-1	36-2	23	ST-23 complex/Cluster A3
577	Y:14,19:P1.-	5-2	10-1	36-2	23	ST-23 complex/Cluster A3
578	Y:14,19:P1.-	5-2	10-1	36-2	23	ST-23 complex/Cluster A3
579	Y:14,19:P1.-	5-2	10-1	36-2	23	ST-23 complex/Cluster A3
580	Y:14,19:P1.-	5-2	10-1	36-2	1655	ST-23 complex/Cluster A3
581	Y:14,19:P1.-	5-2	10-1	36-2	23	ST-23 complex/Cluster A3
582	Y:14,19:P1.-	5-2	10-1	36-2	23	ST-23 Complex/Cluster A3
583	Y:14,19:P1.-	5-2	10-1	36-2	23	ST-23 Complex/Cluster A3
584	Y:14,19:P1.-	5-2	10-1	36-2	23	ST-23 Complex/Cluster A3
585	Y:14,19:P1.-	5-2	10-1	36-2	23	ST-23 Complex/Cluster A3
586	Y:14,19:P1.-	5-2	10-1	36-2	23	ST-23 Complex/Cluster A3
587	Y:14,19:P1.-	5-2	10-2	36-2	3587	ST-23 Complex/cluster A3
588	Y:14,19:P1.-	5-2	10-1	36-2	23	ST-23 Complex/cluster A3
589	Y:14,19:P1.-	5-2	10-1	36-2	10880	ST-23 complex/Cluster A3
590	Y:14,19:P1.-	5-2	10-1	36-2	23	ST-23 Complex/cluster A3
591	Y:14,19:P1.-	5-2	10-1	36-2	23	ST-23 Complex/cluster A3
592	Y:14,19:P1.-	5-2	10-29	36-2	23	ST-23 complex/Cluster A3
593	Y:14,19:P1.-	5-2	10-1	36-2	23	ST-23 complex/Cluster A3
594	Y:14,19:P1.-	5-2	10-1	36-2	23	ST-23 complex/Cluster A3
595	Y:14,19:P1.-	5-2	10-1	36-2	23	ST-23 complex/Cluster A3
596	Y:14,19:P1.-	5-2	10-1	36-2	23	ST-23 complex/Cluster A3
597	Y:14,19:P1.-	5-2	10-1	36-2	23	ST-23 complex/Cluster A3
598	Y:14,19:P1.-	5-2	10-1	36-2	23	ST-23 complex/Cluster A3
599	Y:14,19:P1.-	5-2	10-1	36-2	23	ST-23 complex/Cluster A3
600	Y:14,19:P1.-	5-2	10-29	36-2	23	ST-23 complex/Cluster A3
601	Y:14,19:P1.-	5-2	10-1	36-2	23	ST-23 complex/Cluster A3
602	Y:14,19:P1.-	5-2	10-1	36-2	23	ST-23 complex
603	Y:14,19:P1.-	5-2	10-1	36-2	23	ST-23 complex
604	Y:14,19:P1.-	5-2	10-1	36-2	23	ST-23 complex
605	Y:14,19:P1.-	5-2	10-1	36-2	23	ST-23 complex
606	Y:14,19:P1.-	5-1	10-1	36-2	1655	ST-23 complex
607	Y:14,19:P1.-	5-2	10-1	36-2	23	ST-23 complex
608	Y:14,19:P1.-	5-2	10-1	36-2	10732	ST-23 complex

609	Y:14,19:P1.-	5-2	10-1	36-2	10732	ST-23 complex
610	Y:14,19:P1.-	5-2	10-1	36-2	183	ST-23 complex
611	Y:14,19:P1.-	5-2	10-1	36-2	23	ST-23 complex
612	Y:14,19:P1.-	5-2	10-1	36-2	23	ST-23 complex
613	Y:14,19:P1.-	5-2	10-1	36-2	23	ST-23 complex
614	Y:14,19:P1.-	5-2	10-1	36-2	10466	ST-23 complex
615	Y:14,19:P1.-	5-2	10-1	36-2	23	ST-23 complex
616	Y:14,19:P1.-	5-2	10-1	36-2	23	ST-23 complex
617	Y:14,19:P1.-	5-2	10-1	36-2	23	ST-23 complex
618	Y:14,19:P1.-	5-2	10-1	36-2	10466	ST-23 complex
619	Y:14,19:P1.-	5-2	10-1	36-2	23	ST-23 complex
620	Y:14,19:P1.-	5-2	10-1	36-2	23	ST-23 complex
621	Y:14,19:P1.-	5-2	10-62	36-2	23	ST-23 complex
622	Y:14,19:P1.-	5-2	10-1	36-2	10466	ST-23 complex
623	Y:14,19:P1.-	5-2	10-1	36-2	23	ST-23 complex
624	Y:14,19:P1.-	5-2	10-1	36-2	23	ST-23 complex
625	Y:14,19:P1.-	5-2	10-1	36-2	23	ST-23 complex
626	Y:14,19:P1.-	5-2	10-1	36-2	23	ST-23 complex
627	Y:14,19:P1.-	5-2	10-1	36-2	10466	ST-23 complex
628	Y:14,19:P1.-	5-2	10-1	36-2	10732	ST-23 complex
629	Y:14,19:P1.-	5-2	10-1	36-2	23	ST-23 complex
630	Y:14,19:P1.-	5-2	10-1	36-2	23	ST-23 complex
631	Y:14,19:P1.-	5-2	10-1	36-2	23	ST-23 complex
632	Y:14,19:P1.-	5-2	10-1	36-2	10732	ST-23 complex
633	Y:14,19:P1.-	5-2	10-1	36-2	23	ST-23 complex
634	Y:14,19:P1.-	5-2	10-1	36-2	23	ST-23 complex
635	Y:14,19:P1.-	5-2	10-2	36-2	3587	ST-23 complex
636	Y:14,19:P1.-	5-2	10-1	36-2	10732	ST-23 complex
637	Y:14,19:P1.-	5-2	10-1	36-2	23	ST-23 complex
638	Y:14,19:P1.-	5-2	10-1	36-2	23	ST-23 complex
639	Y:14,19:P1.-	5-2	10-1	new	10466	ST-23 complex
640	Y:14,19:P1.-	5-2	10-1	36-2	23	ST-23 complex
641	Y:14,19:P1.-	5-2	10-1	36-2	23	ST-23 complex
642	Y:14,19:P1.-	5-2	10-1	36-2	23	ST-23 complex
643	Y:14,19:P1.10	5-2	10-12	36-2	23	ST-23 complex/Cluster A3
644	Y:14,19:P1.2,5	5-1	2-2	36-2	23	ST-23 Complex/cluster A3
645	Y:14,19:P1.2,5	5-1	2-2	36-2	23	ST-23 complex/Cluster A3
646	Y:14,19:P1.2,5	5-1	2-2	36-2	23	ST-23 complex
647	Y:14,19:P1.2,5	5-1	2-2	36-2	23	ST-23 complex
648	Y:14,19:P1.5	5-1	10-8	36-2	2880	ST-167 complex
649	Y:14,19:P1.5	5-1	10-4	36-2	11604	ST-23 complex/Cluster A3
650	Y:14,19:P1.5	5-1	10-1	36-2	1655	ST-23 complex/Cluster A3
651	Y:14,19:P1.5	5-1	10-1	36-2	1655	ST-23 complex/Cluster A3
652	Y:14,19:P1.5	5-1	10-1	36-2	1655	ST-23 complex/Cluster A3

653	Y:14,19:P1.5	5-1	10-1	36-2	1655	ST-23 complex/Cluster A3
654	Y:14,19:P1.5	5-1	10-1	36-2	1655	ST-23 complex/Cluster A3
655	Y:14,19:P1.5	5-1	10-1	36-2	1655	ST-23 complex/Cluster A3
656	Y:14,19:P1.5	5-1	10-4	36-2	23	ST-23 complex/Cluster A3
657	Y:14,19:P1.5	5-1	10-1	36-2	1655	ST-23 complex/Cluster A3
658	Y:14,19:P1.5	5-1	10-1	36-2	1655	ST-23 complex/Cluster A3
659	Y:14,19:P1.5	5-1	10-1	36-2	1655	ST-23 complex/Cluster A3
660	Y:14,19:P1.5	5-1	10-1	36-2	1655	ST-23 complex/Cluster A3
661	Y:14,19:P1.5	5-1	10-1	36-2	1655	ST-23 complex/Cluster A3
662	Y:14,19:P1.5	5-1	10-4	36-2	1655	ST-23 complex/Cluster A3
663	Y:14,19:P1.5	5-1	10-1	36-2	1655	ST-23 complex
664	Y:14,19:P1.5	5-1	10-1	36-2	1655	ST-23 complex
665	Y:14,19:P1.5	5-1	10-1	36-2	1655	ST-23 complex
666	Y:14,19:P1.5	5-1	10-8	36-2	2880	ST-167 complex
667	Y:14,19:P1.5	5-1	10-1	36-2	1655	ST-23 complex
668	Y:14,19:P1.5	5-1	10-1	36-2	1655	ST-23 complex
669	Y:14,19:P1.5	5-1	10-8	36-2	2880	ST-167 complex
670	Y:14,19:P1.5	5-1	10-1	36-2	1655	ST-23complex
671	Y:14,19:P1.5	5-1	10-4	36-2	14319	ST-23 complex
672	Y:14,19:P1.6	18-1	3	38	1157	ST-1157 complex
673	Y:15,19:P1.16	21	16	37-1	1466	ST-174 complex
674	Y:15,19:P1.16	21	16	37-1	1466	ST-174 complex
675	Y:15,19:P1.16	21	16	37-1	1466	ST-174 complex
676	Y:15,19:P1.16	21	16	37-1	1466	ST-174 complex
677	Y:15,19:P1.16	21	16	37-1	1466	ST-174 complex
678	Y:19:P1.-	5-2	10-1	36-2	23	ST-23 complex/Cluster A3
679	Y:19:P1.-	5-2	10-1	36-2	23	ST-23 complex
680	Y:19:P1.-	5-2	10-1	36-2	23	ST-23 complex
681	Y:19:P1.-	5-1	10-1	36-2	1655	ST-23 complex
682	Y:19:P1.-	5-2	10-1	36-2	23	ST-23 complex
683	Y:19:P1.-	5-2	10-1	36-2	269	T-269 complex
684	Y:19:P1.-	5-2	10-1	36-2	23	ST-23 complex
685	Y:19:P1.-	5-2	10-62	36-2	23	ST-23 complex
686	Y:19:P1.16	21-7	16	37-1	1157	ST-1157 complex
687	Y:19:P1.16	21	16	37-1	1466	ST-174 complex
688	Y:19:P1.2,5	5-1	2-2	36-2	23	ST-23 complex
689	Y:19:P1.5	5-1	10-4	36-2	3705	ST-167 complex
690	Y:19:P1.5	5-1	10-1	36-2	13454	ST-23 complex
691	Y:19:P1.5	5-1	10-1	36-2	1655	ST-23 complex/Cluster A3
692	Y:19:P1.5	5-1	10-1	36-2	1655	ST-23 complex
693	Y:19:P1.5	5-1	10-1	36-2	1655	ST-23 complex
694	Y:19:P1.5	5-1	10-1	36-2	1655	ST-23 complex
695	Y:19:P1.5	5-1	10-1	36-2	1655	ST-23 complex
696	Y:2a:P1.2,5	5	2	36-2	11	ST-11 complex/37 complex

697	Y:2c:P1.2,5	5-1	2-2	36-2	23	ST-23 complex/Cluster A3
698	Y:2c:P1.2,5	5-1	2-2	36-2	6800	ST-23 Complex/cluster A3
699	Y:2c:P1.2,5	5-1	2-2	36-2	23	ST-23 Complex/cluster A3
700	Y:2c:P1.2,5	5-1	2-2	36-2	23	ST-23 complex/Cluster A3
701	Y:2c:P1.2,5	5-1	2-2	36-2	4183	ST-23 complex/Cluster A3
702	Y:2c:P1.2,5	5-1	2-2	36-2	23	ST-23 complex/Cluster A3
703	Y:2c:P1.2,5	5-1	2-2	36-2	23	ST-23 Complex/cluster A3
704	Y:2c:P1.5	5-1	10-1	36-2	1624	ST-167 Complex
705	Y:2c:P1.5	5-1	10-4	36-2	1624	ST-167 Complex
706	Y:2c:P1.5	5-1	10-1	36-2	1624	ST-167 Complex
707	Y:2c:P1.5	5-1	10-4	36-2	1624	ST-167 Complex
708	Y:2c:P1.5	5-1	10-1	36-2	1624	ST-167 Complex
709	Y:2c:P1.5	5-1	10-4	36-2	9579	ST-167 Complex
710	Y:2c:P1.5,16	5-1	10-1	36-2	1624	ST-167 Complex
711	Y:4:P1.-	5-2	10-1	36-2	23	ST-23 complex
712	Y:NT:P1.-	Deletion	10-4	36-2	1624	ST-167 complex
713	Y:NT:P1.16	21	16	37-1	14272	unassigned
714	Y:NT:P1.2,5	5-1	2-2	36-2	23	ST-23 complex/Cluster A3
715	Y:NT:P1.2,5	5-1	2-2	36-2	23	ST-23 complex/Cluster A3
716	Y:NT:P1.2,5	5-1	2-2	36-2	12163	ST-23 complex/Cluster A3
717	Y:NT:P1.2,5	5-1	2-2	36-2	23	ST-23 complex/Cluster A3
718	Y:NT:P1.2,5	5-1	2-2	36-2	23	ST-23 complex/Cluster A3
719	Y:NT:P1.2,5	5-1	2-2	36-2	23	ST-23 complex/Cluster A3
720	Y:NT:P1.2,5	5-1	2-2	36-2	23	ST-23 complex/Cluster A3
721	Y:NT:P1.2,5	5-1	2-2	36-2	3582	ST-23 complex
722	Y:NT:P1.2,5	5-1	2-2	36-2	1157	ST-1157 complex
723	Y:NT:P1.2,5	5-1	2-2	36-2	3582	ST-23 complex
724	Y:NT:P1.2,5	5-1	2-2	36-2	3582	ST-23 complex
725	Y:NT:P1.2,5	5-1	2-2	36-2	3582	ST-23 complex
726	Y:NT:P1.2,5	5-1	2-2	36-2	23	ST-23 complex
727	Y:NT:P1.2,5	5-1	22	36-2	23	ST-23 complex
728	Y:NT:P1.2,5	5-1	2-2	36-2	3582	ST-23 complex
729	Y:NT:P1.2,5	5-1	2-2	36-2	16114	ST-22 complex
730	Y:NT:P1.2,5	5-1	2-2	36-2	3582	ST-23 complex
731	Y:NT:P1.2,5	5-1	2-2	36-2	3582	ST-23 complex
732	Y:NT:P1.2,5	5-1	2-2	36-2	3582	ST-23 complex
733	Y:NT:P1.5	5-1	10-4	36-2	1624	ST-167 complex
734	Y:NT:P1.5	5-1	10-1	36-2	17796	ST-167 complex
735	Y:NT:P1.5	5-1	10-4	36-2	11603	unassigned
736	Y:NT:P1.5	5-1	10-4	36-2	11847	ST-167 complex
737	Y:NT:P1.5	5-1	10-4	36-2	11847	ST-167 complex
738	Y:NT:P1.5	5-1	10-4	36-2	12967	ST-167 Complex
739	Y:NT:P1.5	5-1	10-4	36-2	1624	ST-167 Complex
740	Y:NT:P1.5	5-1	10-1	36-2	12965	Unassigned

741	Y:NT:P1.5	5-1	10-1	36-2	1655	ST-23 complex/Cluster A3
742	Y:NT:P1.5	5-1	10-1	36-2	6464	unassigned
743	Y:NT:P1.5	5-1	10-1	36-2	6464	unassigned
744	Y:NT:P1.5	5-1	10-1	36-2	6464	unassigned
745	Y:NT:P1.5	5-1	10-1	36-2	1655	ST-23 complex/Cluster A3
746	Y:NT:P1.5	5-1	10-4	36-2	1624	ST-167 Complex
747	Y:NT:P1.5	5-1	10-1	36-2	11603	unassigned
748	Y:NT:P1.5	5-1	10-4	36-2	10908	unassigned
749	Y:NT:P1.5	5-1	10-4	36-2	1624	ST-167 Complex
750	Y:NT:P1.5	5-1	10-4	36-2	1624	ST-167 Complex
751	Y:NT:P1.5	5-1	10-4	36-2	1624	ST-167 complex
752	Y:NT:P1.5	5-1	10-4	36-2	10908	unassigned
753	Y:NT:P1.5	5-1	10-4	36-2	3980	ST-167 Complex
754	Y:NT:P1.5	5-1	10-4	36-2	3980	ST-167 complex
755	Y:NT:P1.5	5-1	10-4	36-2	3980	ST-167 complex
756	Y:NT:P1.5	5-1	10-4	36-2	10908	unassigned
757	Y:NT:P1.5	5-1	10-4	36-2	3980	ST-167 complex
758	Y:NT:P1.5	5-1	10-4	36-2	9579	ST-167 Complex
759	E:15:P1.2,5	5	2	36-2	60	ST-60 complex
760	E:19:P1.-	21-7	16-75	37-1	1157	ST-1157 complex
761	E:NT:P1.5	5-1	10-26	36-2	12522	unassigned
762	Z:17:P1.15,19	19	15-39	36	6502	ST-2507 complex
763	Z:NT:P1.7	7	30	38	103	ST-103 Complex
764	NE:15:P1.6	18	25-1	38-1	198	ST-198 complex
765	NE:15:P1.6	18	25-1	38-1	198	ST-198 complex
766	NE:15:P1.9	17-4	9	35-1	823	ST-198 complex
767	NE:17:P1.13	7-2	13-9	35-1	11732	unassigned
768	NE:4:P1.15,19	19	15	36	34	ST-32 complex
769	NE:4:P1.6	18	25	38-1	34	ST-32 Complex/ET-5 Complex
770	NE:NT:P1.-	7-2	30-2	38	53	ST-53 Complex
771	NE:NT:P1.-	18-1	30-3	38	1117	ST-1117 complex
772	NE:NT:P1.-	7-2	30-9	38	53	ST-53 complex
773	NE:NT:P1.14	7-2	14	36	4940	ST-269 complex
774	NE:NT:P1.7	7	30	38	53	ST-53 complex

Antigenic formula = serogroup/serotype/serosubtype

Serogroup NE = non-encapsulated

Supplementary Table S6.

Characteristics of culture-confirmed invasive meningococcal disease (IMD) cases in Canada, 2015 to 2023 by serogroups*.

Number of IMD cases by serogroup

Age groups	MenB	MenC	MenY	MenW
< 5 year	89 (30.5%)	5 (10.9%)	7 (3.7%)	36 (15.7%)
5-14 years	20 (6.8%)	0 (0%)	7 (3.7%)	7 (3.0%)
15-24 years	59 (20.2%)	4 (8.7%)	46 (24.5%)	31 (13.5%)
25-39 years	31 (10.6%)	9 (19.5%)	16 (8.5%)	27 (11.7%)
40-59 years	35 (12.0%)	12 (26.1%)	35 (18.6%)	47 (20.4%)
≥ 60 years	58 (19.9%)	16 (34.8%)	77 (41.0%)	82 (35.7%)
Unknown	0	0	0	2
All age groups	292	46	188	232
Age range	6 days to 92 years	1 day to 88 years	3 months to 97 years	24 days to 97 years
Median age	20.0 years	48.0 years	52.0 years	48.0 years
Mean age	29.1 years	45.3 years	48.3 years	43.0 years
Females	132	25	100	128
Males	159	20	88	101
Female/Male	0.83	1.25	1.14	1.27
Blood	195	29	155	211
CSF+	75	9	15	10
Blood/CSF	14	1	8	6
Joint	3	5	6	4
Joint/Blood	1	1	0	0
Others	4 (3 brain/1 ascite)	1 (pericardial fluid)	4 (lung, vitreous, peritoneal, and pericardial fluids)	1 (pericardial fluid)

* Serogroups: serogroup B (MenB); serogroup C (MenC); serogroup Y (MenY); serogroup W (MenW).
+ CSF = cerebrospinal fluid

Supplementary Figure 1.

Canada with provincial and territorial names, 2022, from the Atlas of Canada online (natural-resources.canada.ca/maps-tools-and-publications/maps/atlas-canada/reference-maps/26120). This reproduction is a copy of an official work published by the Government of Canada, and it was not reproduced in affiliation with said entity or with its endorsement.

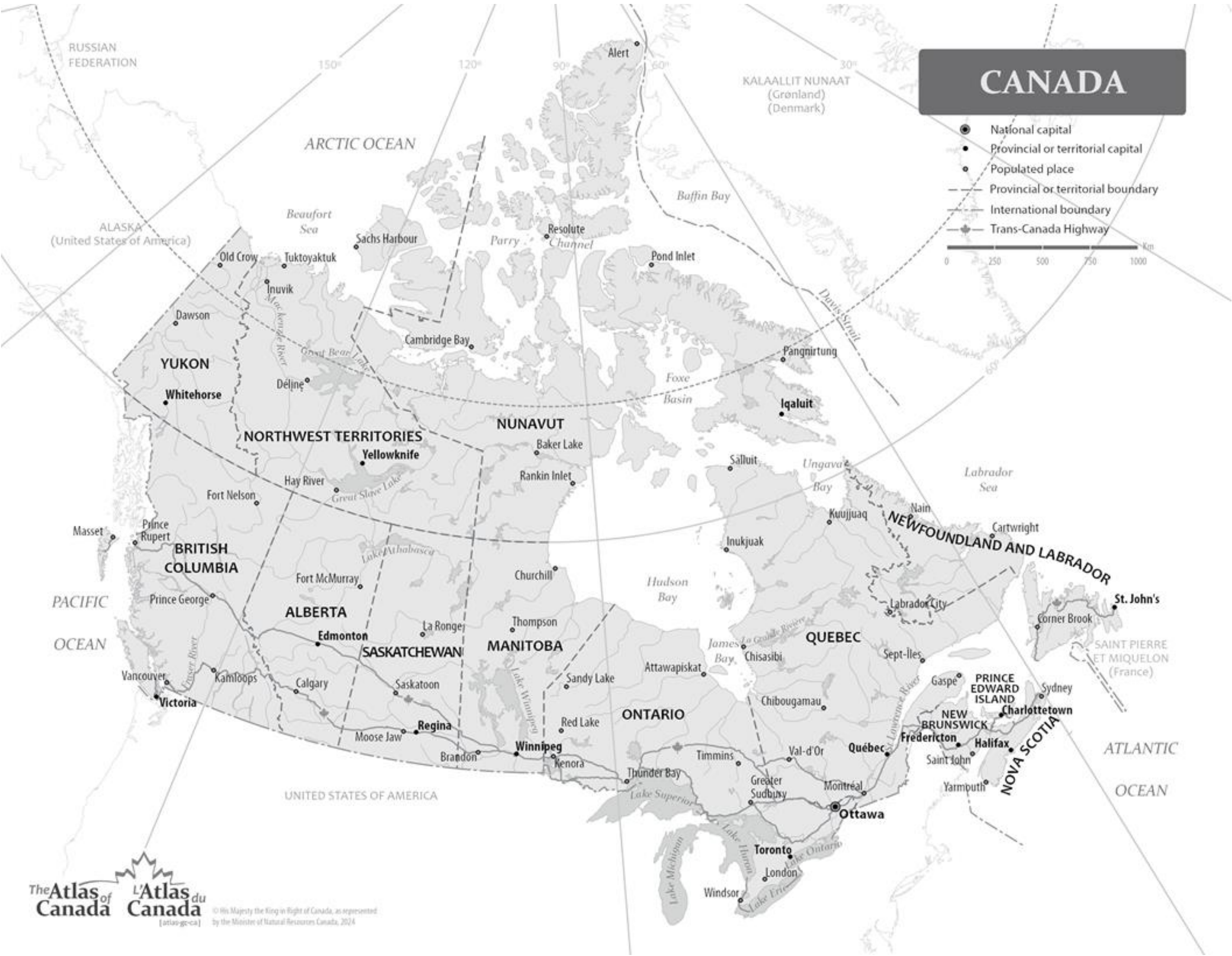

Supplement: Uncited Supplementary Material 1. [file jmm-74-01979-s001.pdf]
